# Deep Learning of Nanopore Sensing Signals Using a Bi-Path Network

**DOI:** 10.1021/acsnano.1c03842

**Published:** 2021-08-17

**Authors:** Dario Dematties, Chenyu Wen, Mauricio David Pérez, Dian Zhou, Shi-Li Zhang

**Affiliations:** † Instituto de Ciencias Humanas, Sociales y Ambientales CONICET Mendoza Technological Scientific Center, Mendoza M5500, Argentina; ‡ Division of Solid-State Electronics, Department of Electrical Engineering, Uppsala University, SE-751 03 Uppsala, Sweden; § Department of Electrical and Computer Engineering, University of Texas at Dallas, Richardson, Texas 75080, United States

**Keywords:** neural network, deep learning, nanopore sensors, pulse-like signals, feature extraction

## Abstract

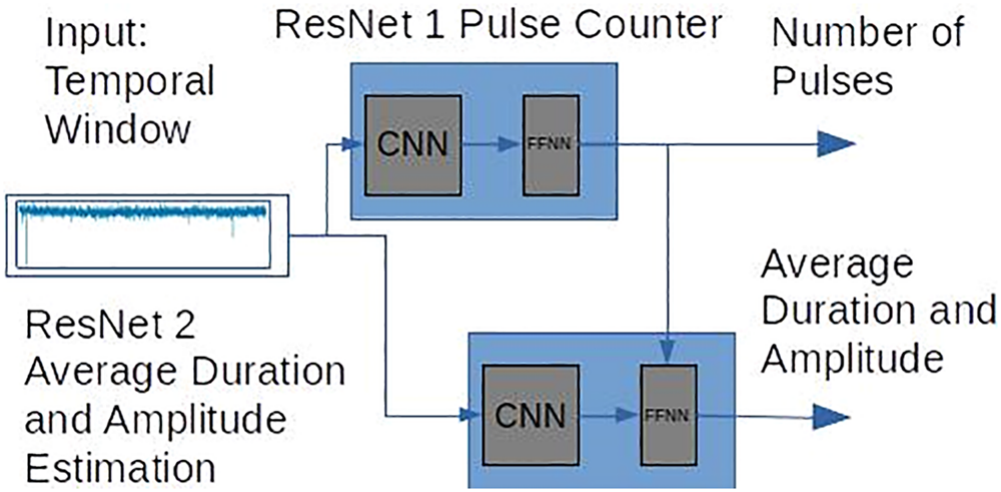

Temporal changes in electrical resistance of a nanopore sensor caused by translocating target analytes are recorded as a sequence of pulses on current traces. Prevalent algorithms for feature extraction in pulse-like signals lack objectivity because empirical amplitude thresholds are user-defined to single out the pulses from the noisy background. Here, we use deep learning for feature extraction based on a bi-path network (B-Net). After training, the B-Net acquires the prototypical pulses and the ability of both pulse recognition and feature extraction without *a priori* assigned parameters. The B-Net is evaluated on simulated data sets and further applied to experimental data of DNA and protein translocation. The B-Net results are characterized by small relative errors and stable trends. The B-Net is further shown capable of processing data with a signal-to-noise ratio equal to 1, an impossibility for threshold-based algorithms. The B-Net presents a generic architecture applicable to pulse-like signals beyond nanopore currents.

Nanopore sensing technology finds a wide scope of applications exemplified with DNA sequencing,^1^ protein profiling,^2^ small chemical molecule detection,^3^ and nanoparticle characterization.^4^ When analytes pass through a nanopore, characteristic pulses or spikes are generated on monitoring current traces.^5^ Properties of the analytes including size, shape, concentration, charge, and dipole moment can be inferred from the amplitude, width (duration), frequency, and waveform of such spikes.^2,4,6,7^ Traditional procedures in several different variants to recognize and extract translocation events, *i*.*e*., spikes, from noisy current traces are typically based on a user-defined amplitude threshold as a criterion to separate the spikes from background noise fluctuations.^8,9^ The flow of data processing for nanopore signals, as well as related algorithms, is a widely accepted establishment (see Note 1 of the Supporting Information (SI)). The determination of spikes is, thus, highly dependent on how the threshold is defined. There is apparently risk that this approach becomes subjective. Although progress has been made in diminishing the subjectivity with empirical selection of the threshold, *e*.*g*., defining the threshold by referring to the background noise level, user intervention cannot be totally avoided.^10^ Thus, conventional techniques for extracting features from raw data have been historically limited by their capacity. Machine learning based algorithms have, therefore, been considered for processing the nanopore sensing data. To isolate the translocation spikes from the background noise, a hidden Markov model has been adopted to discriminate the open-pore state with a relatively high current level from the blockage state in the spike form with a lower current level. The spikes associated with the current blockade events, as well as their basic features, can, then, be extracted automatically.^11,12^ In addition, neural network based deep learning (DL) algorithms have been adapted to the nanopore sensing data of one-dimensional nature. A convolutional neural network (CNN) has been developed to identify the fine patterns in the current blockage state that are caused by a sequence of tailor-designed hairpin loops placed at predefined positions as the barcode of the target DNA.^13^ However, these developments target specific applications and lack the universality for general translocation spike signals. Although several other DL-based algorithms, such as CNN, feed-forward fully connected neural network (FFNN), and long short-term memory, have also been used, most of them have a destined function as a classifier to distinguish different kinds of analytes by the features of their translocation spikes.^14,15^ They are, hence, incapable of spike reorganization and feature extraction that this work is set to achieve. Specifically, an advanced algorithm based on a DL architecture in the form of a bi-path network (B-Net) is proposed in this work. The B-Net is capable of directly transforming raw data into an appropriate representation, from which certain ending subsystems, such as a classifier, can detect patterns in the input. In other words, the three important features of the spikes, *i*.*e*., amplitude, duration, and frequency, can be extracted as a package solution covering the demands for an appropriate nanopore sensing technology.

The B-Net is based on a highly consolidated DL architecture, the residual neural network (ResNet), as depicted in Figure 1. As an integral part of the ResNet architecture, the CNN can be utilized in any dimensional space, although dimensions up to three are the most commonly used depending on the application. For example, three-dimensional CNNs are normally used for volumetric data in medical imaging.^16^ On the other hand, two-dimensional CNNs are the most popular for images and matrices.^16^ As is the case of the B-Net introduced here, one-dimensional CNNs have also been used for signals and time series such as automated atrial fibrillation detection^17^ and sleep arousal detection.^18^

**Figure 1:**
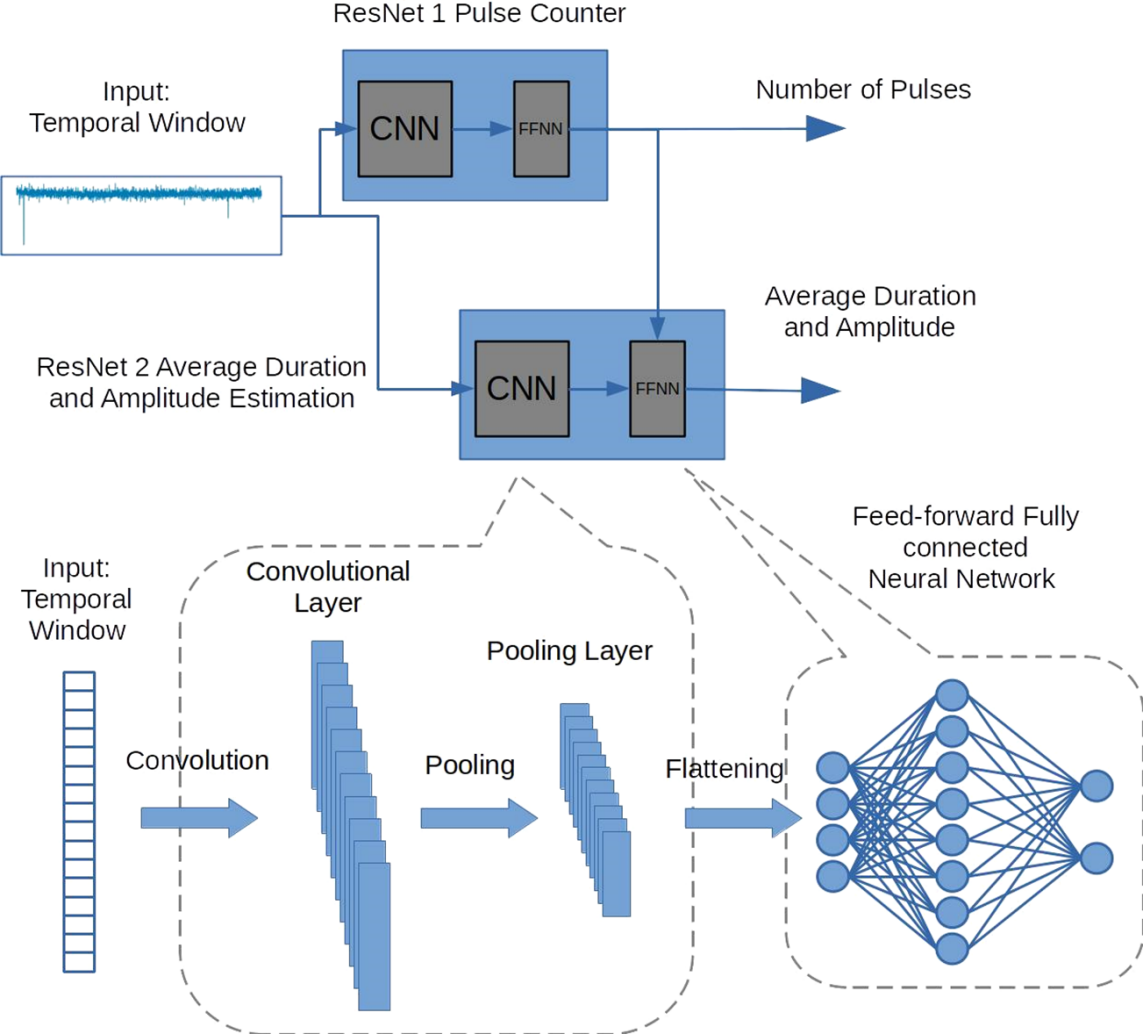
Architecture of the B-Net. This network features a two-way architecture with two ResNets. Each ResNet consists of a CNN and an FFNN. ResNet 1 predicts the number of pulses or translocation events in a temporal window. ResNet 2 forecasts the average translocation amplitude and duration of all the pulses within the same window. ResNet 1 also feeds its output, as an internal input, to the FFNN of ResNet 2. The convolutional section in our implementation has been adapted to processing one-dimensional data. The fully connected architecture, on the other hand, outputs real valued predictions such as average amplitude and duration of the translocation events in a temporal window.

The B-Net is first evaluated on artificially generated data sets. It is, then, employed for experimental data of λ-DNA and *streptavidin* translocation in solid-state nanopores. Compared to its traditional algorithm counterparts, the B-Net shows improved performance from the perspective of robustness, objectivity, and stability. It is anticipated that the developed B-Net, with necessary adjustment and optimization, is applicable to feature extraction of other pulse-like signals, *e*.*g*., transverse tunneling current for single-molecule detection, spikes from neural cells and systems, and electrocardio pulses. It should be clarified that the B-Net concept is designed for handling pulse-like signals. Although DL algorithms have been widely used for treating DNA/RNA sequencing data generated from a nanopore sequencer, the B-Net in its present form is not meant for that. The sequencing data possess essentially different informative features usually as random stepwise jumps among different levels. Processing such sequencing data belongs to a totally different field.

## Results/Discussion

### Neural Network Architecture

The holy grail in all DL architectures is depth. The rationale behind these successful methods is built on experimental evidence, which suggests that adding more layers to a deep neural network (DNN) provides us with more abstract output vectors that would better represent the hidden features from raw input data. Such rich features would finally allow us to better perform the task for which the network is trained. Yet, this desirable phenomenon has important limitations since adding more layers to a network comes with penalties, *e*.*g*., vanishing and exploding gradients^19,20^ that are often encountered when training artificial neural networks with gradient-based methods. In each training iteration, such methods update the weights of the neural network proportionally to the partial derivatives of error functions with respect to the given weights. In an *n*-layer network, a small/large gradient could end up with becoming vanishingly small/explosively large in early layers of the pipeline, provided that the gradient decreases/increases exponentially with *n*. This undesired positive feedback can prevent weights from properly updating their values and potentially impeding further improvement of the neural network. ResNet is adopted in the B-Net to mitigate such risks. ResNet is an artificial DNN that uses CNNs and skips connections or bypasses some layers.^21^ The main motivation for bypassing/skipping layers is to avoid the problem of vanishing/exploding gradients. The architecture of the B-Net is visualized in Figure 1. It is a bi-path architecture composed of two parallel ResNets. Both ResNets receive the same input that is simply a temporal window, segmented from a complete nanopore translocation current trace. They also return the predicted number of pulses and average amplitude and duration of such pulses in the window.

The window width should be carefully selected according to the translocation frequency in the target data sets. The window should not be too small such that most of the windows are blank without any spike, which may cause an imbalance during the training process. The window should not be too large to include an excessive number of spikes either because some detailed information may become compromised in the averaged values over one window as the output. Although averaging in each window to obtain the feature values may compromise revealing some details of individual events, our algorithm is capable of capturing the statistical properties, *e*.*g*., mean, standard deviation, and distribution function. Most of the commonly used methods are based on the statistical properties of features, instead of the features of single events, to infer the physical parameters of the analytes or to identify/classify the analytes. In contrast, our algorithm advantageously uses the averaged feature values as the output, and the fineness of the results can be tuned by varying the window width. Furthermore, an autocorrelation procedure for window selection based on a few prior tests can be easily implemented to retain the objectivity of the B-Net approach.

As shown in Figure 1, ResNet 1 is assigned to predict the number of translocation spikes (pulses) in the window, while ResNet 2 forecasts the average amplitude and duration of all the pulses found in the same window. ResNet 1 also provides its output as an internal input for the FFNN in ResNet 2. An additional description of the B-Net architecture is provided in Methods and Note 2 of the SI.

### Training and Validation

ResNet 1 and 2 in Figure 1 were implemented by utilizing another DNN, ResNet 18 in the Pytorch DL framework (https://www.pytorch.org).^22^ Since ResNet is originally designed for image processing in two dimensions, necessary modifications were implemented on the architecture to adapt it for the one-dimensional data. Additionally, the last linear layer was replaced by a multilayer perceptron with two layers, and the necessary adaptations were adopted to feed an extra internal input from the ResNet 1 output into the ResNet 2 FFNN, in its final layer. Finally, some batch normalization mechanisms in certain layers of the network were replaced by the group normalization strategy. Our implementation is publicly available.^23^

The B-Net was trained and validated using artificially generated data. It is worth noting that the generated data sets are physics-based involving a set of well-established physical models for nanopore-based sensors. They include the nanopore resistance model,^24^ spike generation model,^25^ and noise model,^26^ by entailing stochastic variations of corresponding parameters in accordance with the related physical mechanisms (Methods and Note 3 of the SI). The B-Net was subsequently evaluated on and applied to both artificial and experimental data (Methods and Note 4 of the SI). Further, five different instances of the network were trained for five different signal-to-noise ratios (SNRs) in the artificial data sets. In all cases, smooth *l*_1_-loss and stochastic gradient descent (SGD) optimization were adopted. Additional details about training, such as batch sizes, learning rate schedules, number of epochs, time consumed, curves of loss, and errors for all B-Net instances in this work can be found in Note 5 of the SI.

### Features Extracted from Generated Data Sets

**Figure 2:**
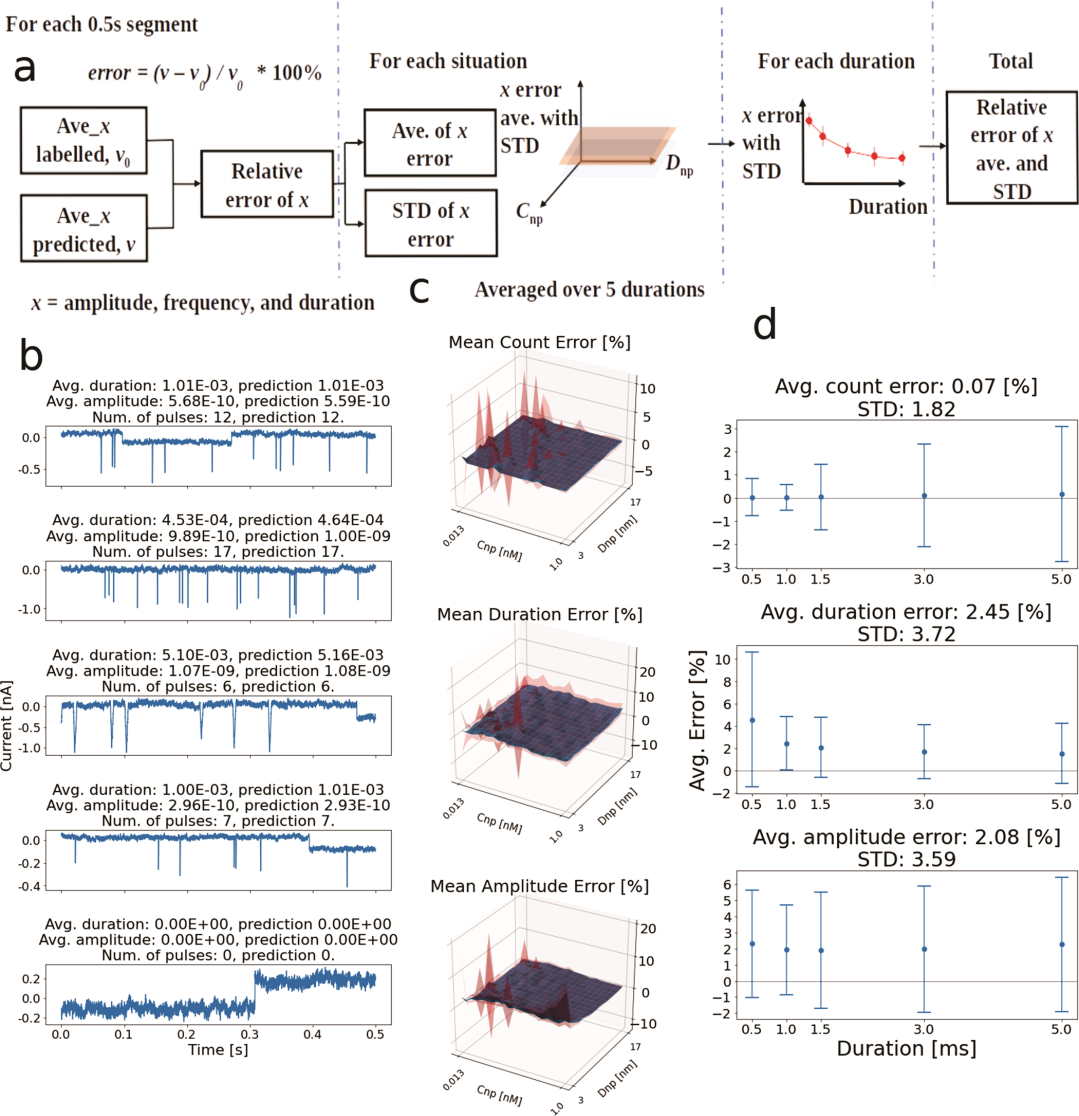
General output of the B-Net and evaluation of the artificially generated test data set with SNR = 4. (a) Complete procedure of performance evaluation of the B-Net. First, the relative errors between ground-truth values and predictions of the B-Net are calculated. Second, the mean error and STD are computed throughout the duration. Third, the average error and STD for each duration are calculated, and finally the total average error and STD are obtained. (b) Example of five trace windows. The ground-truth feature values and the predictions of the B-Net are listed above each window. (c) Surface plots showing the relative errors and their STDs for each situation *vsC*_np_ and *D*_np_, averaged throughout the duration. (d) Average relative errors and STDs for each duration. Final values with the total average errors and STDs are given above each figure.

The general output of the B-Net and the process of performance evaluation are depicted in Figure 2. The B-Net receives a temporal window from a signal trace and returns the predicted average amplitude and duration of all the pulses as well as the number of pulses in the window (Figure 1). Typical windows with the ground truth are shown in Figure 2b along with the resultant predicted average values of amplitude and duration as well as the count of pulses for artificially generated traces with SNR = 4.

### Performance Evaluation

The procedure to performance-evaluate the B-Net is shown in Figure 2a (more details in Methods and Note 4 of the SI). The test data set contains traces for different configurations (Figure 2b) at specified values of concentration of nanospheres (*C*_np_, correlated with translocation frequency), diameter of nanospheres (*D*_np_, correlated with spike amplitude), and translocation dwell time (correlated with duration). The relative error of each data window is calculated for each parameter (*i*.*e*., number of spikes, amplitude, and duration) referred to the ground truth recorded during the data generation, following the formula shown in Figure 2a (details in Methods). Then, the relative errors are averaged throughout the duration in each situation of *C*_np_ and *D*_np_ as displayed in Figure 2c. The blue surface corresponds to the mean value, while the translucent red spike-like surfaces above and beneath the error depict the standard deviation (STD) of the error. The average errors and STDs for each duration in the data set are plotted in Figure 2d. Finally, the total average errors and STDs are computed and shown above each plot (complete evaluation results in Note 4 of the SI).

**Figure 3:**
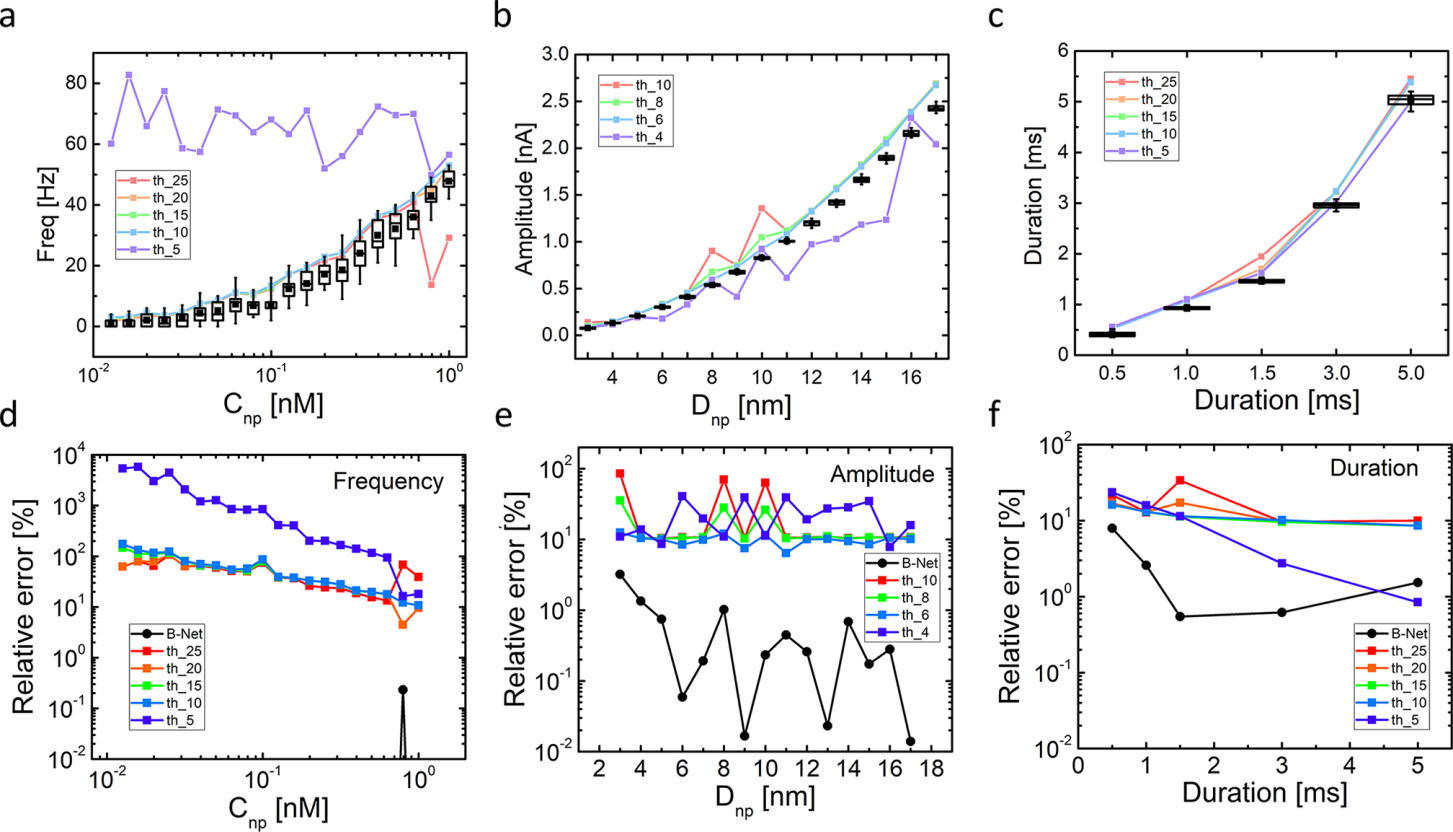
Comparison between the B-Net and the traditional algorithm on the artificially generated test data set with SNR = 4. (a–c) Box charts for variation of spike frequency, amplitude, and duration for translocating nanospheres with different *C*_np_, *D*_np_, and duration extracted using the B-Net (black symbols). They are directly compared with average values for spike frequency, amplitude, and duration extracted using the traditional algorithm with several different *n* values in *th_n* (dot-on lines). Relative errors (whose expressions are given in Methods) for spike frequency, amplitude, and duration predictions by the B-Net and the traditional algorithm are compared in (d)–(f).

The outputs and relative errors of the B-Net are compared to those of the traditional algorithm in Figure 3. Different values of *n* in *th_n* denote the user-defined thresholds of the amplitude as a criterion to distinguish true translocation-generated spikes from fluctuations (noise) in a current trace. Here, *th* stands for threshold and *n* is defined as the number of multiples of the peak-to-peak value of the background noise (more details of the implementation of the traditional algorithm in Methods and SI). The comparison has a focus on processing the artificially generated test data set with SNR = 4.

The B-Net and the traditional algorithm agree well in yielding similar variations of amplitude, frequency, and duration (Figure 3a–c). Here, frequency is the ratio of the number of pulses predicted by the B-Net and the time extension of a temporal window (0.5 s). This computation was repeated for each window in the data set where the statistical features were collected. For the traditional algorithm, frequency is defined as the reciprocal of the time interval between the current and the immediate next event.^10^

The average values of frequency, amplitude, and duration features resulting from the traditional algorithm obviously depend on the selection of the amplitude threshold, as shown by the deviations among the dot-on lines in the figures. Only if the threshold is properly selected can the traditional algorithm return a reasonable result. Small thresholds can lead to assignment of fluctuations as translocation spikes, thereby incorrectly increasing frequency count and lowering both amplitude and duration numerical values. On the other hand, large thresholds can give rise to missing translocation spikes, rendering misleadingly high average amplitude and duration. Furthermore, the feature extraction resulting from the traditional algorithm is clearly less stable than that produced from the B-Net. For example, two small peaks appear in the *th_10* curve at *D*_np_ = 8 and 10 nm in Figure 3a. Such instability arising from the oversensitivity of the traditional algorithm to data generation is obviously absent with the B-Net approach.

The prediction errors for the different features for both algorithms, the B-Net and the traditional, are compared in Figure 3d–f. Except for the prediction of duration in the duration = 5 ms case (Figure 3f), relative errors of the traditional method are notoriously larger than those of the B-Net. Even in the exception, the error of the B-Net is still quite competitive. Strikingly, the relative errors of the B-Net for frequency count are 4 orders of magnitude smaller than those of the traditional algorithm for most C_np_ values (Figure 3d). For amplitude (Figure 3e) and duration (Figure 3f), the relative errors of the B-Net are up to 3 and 2 orders of magnitude, respectively, lower than those of the traditional algorithm. In addition, the relative errors for the traditional algorithm are again highly dependent on the selection of the threshold, confirming the subjectivity of the algorithm. Comprehensive comparisons of frequency, amplitude, and duration for each data set, as well as their relative errors (Note 6 of the SI) affirm the observations above.

### Signal-to-Noise Limitation

**Figure 4:**
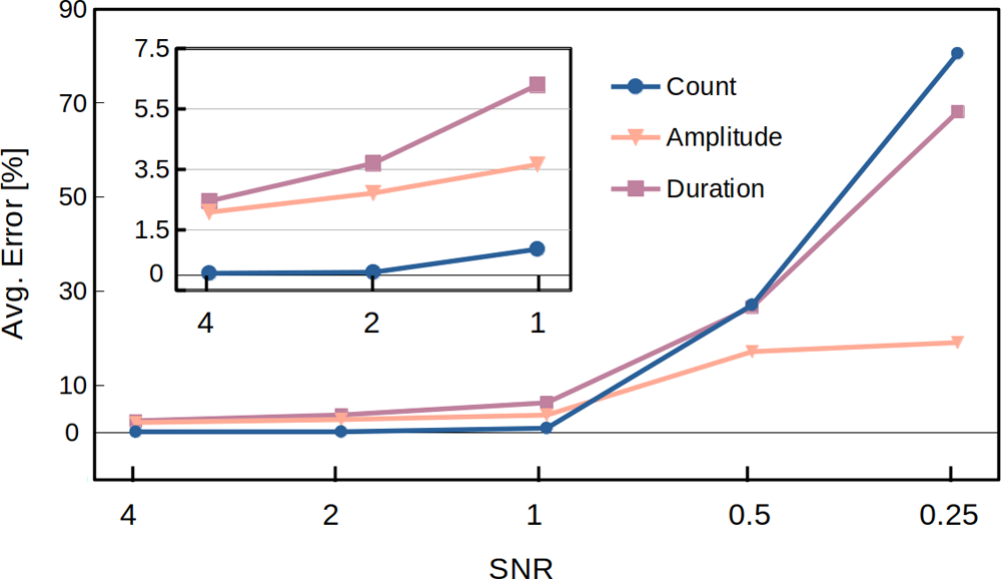
Total average relative errors of frequency, amplitude, and duration *vs* SNR for the B-Net.

To explore the capability of the B-Net in identifying signals from a noisy background of various noise levels, five different networks with the same architecture based on ResNet 18 were trained by data sets with SNR = 4, 2, 1, 0.5, and 0.25. The performance of the B-Net in response to the different noise levels is displayed in Figure 4 (complete evaluation in Note 4 of the SI). Error values represent the relative errors of the B-Net in predicting the number of pulses (count) and the average amplitude and duration of all pulses in the temporal windows. As expected, errors increase with decreasing SNR. Inferred from the steepest slope for the count error in Figure 4, ResNet 1, predicting number of pulses, is more affected than ResNet 2, predicting amplitude and duration, by the level of noise. Similarly, the duration prediction is more susceptible than the amplitude counterpart.

The smallest error for count is produced by ResNet 1 with an average error of 0.067% for SNR = 4. On the other hand, ResNet 2 produces an error of 2.1% and 2.5% in its prediction of amplitude and duration, respectively. For SNR = 1, ResNet 1 is still the best performing part of the B-Net, with an average error of 0.86% for count, while ResNet 2 presents 3.7% and 6.3% of error for amplitude and duration, respectively. The performance starts to degrade severely for SNRs below 1 with errors above 27% (count) for ResNet 1 and above 17% (amplitude) and 26% (duration) for ResNet 2.

The performance of the B-Net is compelling, given that it is almost impossible for the traditional algorithm to correctly recognize translocation spikes in a noisy background with a noise level having a similar amplitude to the spikes, *i*.*e*., SNR = 1. Therefore, this capability grants the B-Net the potential to process data from high-bandwidth systems that are usually characterized by high noise levels. By employing larger architectures in combination with labeled generated data sets that are closer to real measured data, it is possible to further reduce errors and facilitate correct processing of signals with even smaller SNRs.

In the B-Net, the features of spikes are acquired by the algorithm during the training process. They contain as much of the original information as possible, indicating not only picking up obvious features considered in the traditional algorithm, *e*.*g*., amplitude and duration, but also noticing details in the pulses, *e*.*g*., the waveform. These extra features assist the B-Net to better appreciate the difference between a real translocation spike and a background noise peak, even when the two have the same amplitude with SNR = 1. Furthermore, the decision process is flexible and probabilistic, indicating a powerful method with robust performance. As the entire process minimizes the participation and intervention of users, it warrants maximum objectivity; see below.

### Objectivity Analysis of Experimental Data

**Figure 5:**
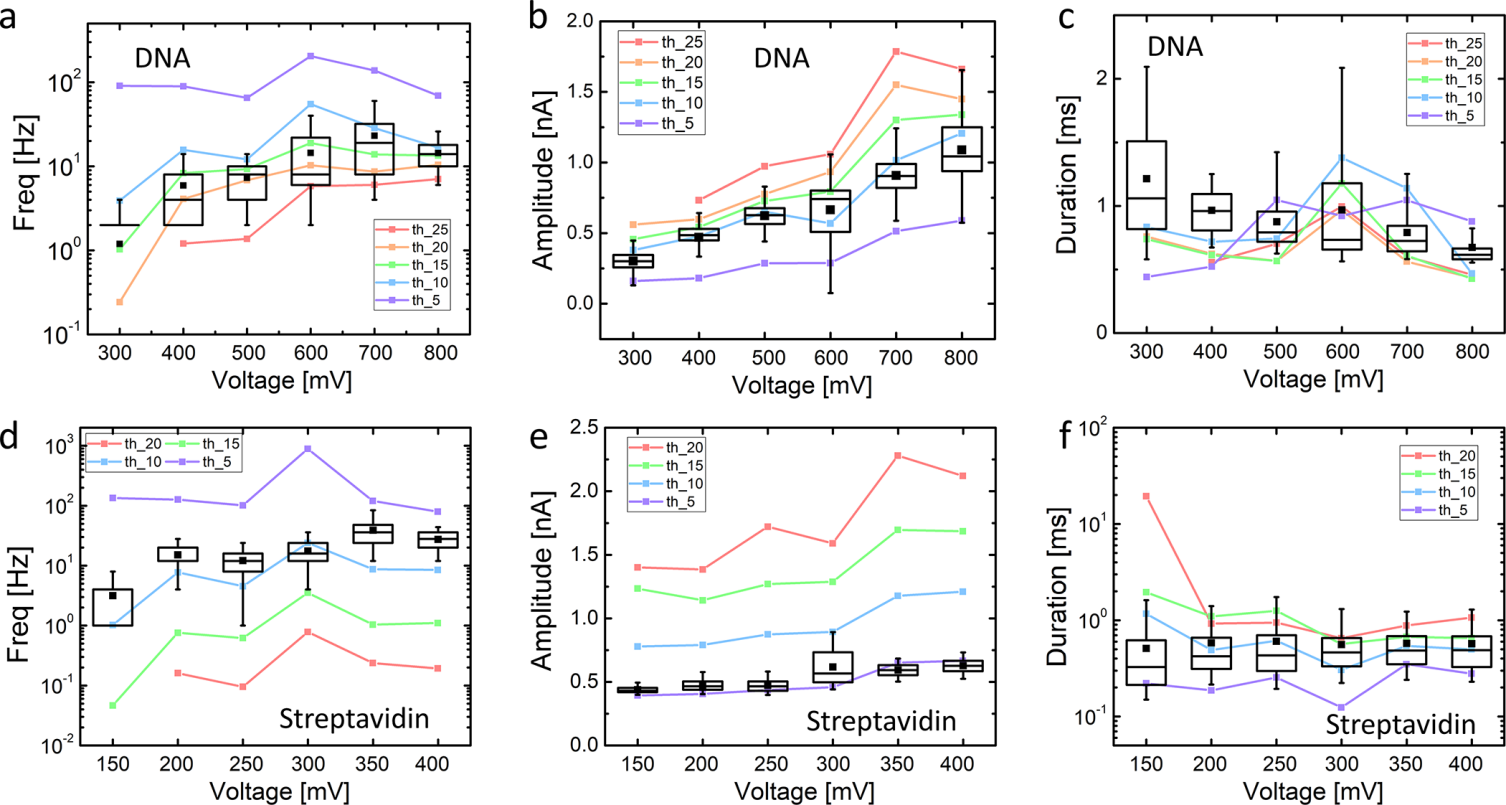
Signal processing of experimental data involving DNA and protein. Variation of spike frequency, amplitude, and duration of λ-DNA (a–c) and *streptavidin* (d–f) translocation with bias voltage. The box charts show the results from the B-Net, while the dot-on lines represent the corresponding results from the traditional algorithm with different thresholds.

The B-Net is also applied to two experimental data sets for nanopore translocation of λ-DNA and *streptavidin* from our previous work.^10^ The outputs produced by the B-Net and those produced by the traditional algorithm are compared in Figure 5a–c for the λ-DNA data set. There is a clear correlation regarding the variation in different features returned by both algorithms. For the B-Net outputs, both frequency and amplitude increase, while the duration decreases with bias voltage. Data analysis shows a linear relationship for all (Figure S20 of the SI), which, to the first-order, is physically plausible. In detail, the linear dependence of amplitude on bias voltage (Figure 5b and Figure S20a) agrees with earlier reports.^27,28^ This is an expected relationship since for a blockade resistance generated by analyte translocation, a higher voltage induces proportionally a larger current. The translocation frequency also displays a linear dependence on voltage (Figure 5a and Figure S20b), which indicates that the analyte capture probability is limited by diffusion, instead of by the energy barrier of the nanopore since the latter would give rise to an exponential dependence on voltage.^29^ It has been shown experimentally that long double-stranded DNA (<10^4^ bps), such as λ-DNA, usually displays a diffusion-limited capture behavior,^30^ in agreement with our finding here. Furthermore, a linear decrease of duration with increasing voltage (Figure 5c and Figure S20c) is simply a result of increased electric field giving rise to a proportionally increased rate of translocation with an invariant ionic mobility, because the DNA translocation is electrophoretically governed.^31^

However, significantly larger dispersions and fluctuations in the output values are evident with the traditional algorithm whose outputs heavily depend on the subjective threshold determined by the user. The deviation among the results from the traditional algorithm with different thresholds is more significant than that for the generated data (Figure 3a–c). Hence, the features extracted from the traditional algorithm lose their objectivity to a certain extent.

A similar comparison for *streptavidin* translocation (Figure 5d,e and Note 7 of the SI) displays also a clear correlation of the variation of the outputs with bias voltage from both algorithms. Compared with the λ-DNA translocation data, the dependence of amplitude and duration on bias voltage is weaker though also linear, in agreement with other reports.^7,32^ The duration is insensitive to bias voltage, which could be related to the limited bandwidth at 10 kHz of the amplifier for data acquisition. When the translocation time is close to or shorter than the time constant defined by the cutoff frequency of the amplifier, the difference in the width of pulses is often smeared out.^33,34^ In addition, the long-duration events primarily caused by the interaction between *streptavidin* and the nanopore are easy to pick up by the readout electronics. They may contribute certain populations to the total events. However, such interaction-caused spikes are independent of bias voltage. According to the simple physical model of diffusive transport,^34^ the translocation frequency is estimated to be 193 Hz. The extracted frequency from the B-Net corresponds to 5–20% of the theoretical value, which is a reasonable ratio in agreement with other reports^34^ (see Note 8 of the SI for details). Furthermore, the dispersion presented by the traditional algorithm is worse than the one with the λ-DNA translocation data. This difference can be related to a lower SNR of the *streptavidin* translocation data. It is shown in Figure 5d,e that the frequency and amplitude predicted by the traditional algorithm are even more dependent on the choice of subjective voltage threshold.

## Conclusions

The analyses and comparisons in preceding sections confirm that the B-Net meets the essential and critical requirements of being objective and avoiding subjective parameters determined by the user. The adverse effects of a subjective and often blind input parameter adjustment are clearly appreciated in Figures 3 and 5, where the predictions of the traditional algorithm sensitively depend on a threshold adjusted beforehand. In contrast to the traditional algorithm replying on user-defined input parameters, the advantages of the B-Net lie also in its clear, stable, and consistent predictions as well as negligibly small relative errors. All this is indicative of the robustness of the B-Net in being able to analyze noisy data thereby easing the otherwise strict demands on the control of experimental conditions. The impressive performance of the B-Net in singling out pulses from a noisy background with relative errors below 1% for count and 5% for amplitude and duration for input data of SNR = 1 provides a validating example. Such a performance is not anticipated for traditional algorithms since when thresholds are used for recognizing translocation spikes, a noise peak, with similar amplitude, can easily be misclassified as a spike originated from a translocation event. Furthermore, the bi-path architecture in the B-Net assigns different categories of tasks (*i*.*e*., pulse count and average feature predictions) to distinct network branches, while the information processed in one branch (*i*.*e*., the pulse counter) is used by the other to predict extra average features. This strategy is naturally in agreement with the architecture of the human brain.^35^

Regardless of all favorable features reviewed above, the B-Net is a DL-based method. As such, it is inherently a data-hungry strategy that works better when there are thousands, millions, or even billions of training examples.^36^ In problems with limited data sources, DL is not an ideal solution. In the specific area of concern, real traces collected from nanopore translocation experiments could be abundant, but they are not labeled. Recruiting staff for labeling such data is not viable given the extensiveness of the data sets needed to train the B-Net. Instead, we have generated our own artificial data sets in this work in order to train, validate, and, then, put in use the B-Net. The comparison experiments conducted against the traditional approaches by processing experimental traces clearly demonstrate that our generated data sets retain a high statistical correlation with the experimental traces collected in the laboratory. Yet, it is imperative to clarify that beyond such favorable results it is impossible to perfectly mimic the statistical distribution immersed in real traces. General palliative methods to solve this problem are available to DL. There are pretraining stages of the networks with which looser requirements are demanded, and smaller labeled data sets can later be used to fine-tune them. Pretraining could be tackled using alternative generated labeled data sets. In this work, we have shown the relevance of the data sets generated to train our B-Net. Our artificially generated data sets can also be used as a pretraining resource in the B-Net. Afterward, adding labels to a much smaller data set of traces collected in the laboratory can be a much more viable endeavor. This could result in a highly qualified network, fine-tuned with real traces. Such a network would show prominent performance differences from a network trained only using artificially generated data sets as the one introduced in this work.

In conclusion, our B-Net algorithm is highly flexible to the input signal and it is not limited to signals from nanopore sensors. A myriad of pulse-like signals, found in biotechnology, medical technology, physical sciences and engineering, information and communication technology, environmental technology, *etc*., can be processed by implementing the robust and objective B-Net. Therefore, the B-Net is a generalizable and flexible platform owing to the flexibility of DL strategies.

## Methods/Experimental

### Data Preparation

#### Artificial Data Generation

The artificially generated data are composed of three parts: (1) randomly appearing translocation spikes, (2) background noise, and (3) baseline variations. The baseline current level, *i*.*e*., the open-pore current, is determined using the resistance model,^24^ with given geometry properties, electrolyte concentration, and bias voltage (more details in Note 3 of the SI). In this system, differently sized nanospheres are used to represent analytes. In the signal generation program, the sampling rate is set at 10 kHz to determine the time step of the signal. According to our previous work,^37^ the probability of appearance of translocation spikes at each time step is correlated to the concentration of nanospheres. The amplitude of spikes is assigned by our translocation model based on the resistance change by steric blockade during the translocation.^38^ The waveform of translocation spikes is approximated using an asymmetrical triangle with adjustable ramping slopes (details in Note 3 of the SI).

According to the related studies, colored Gaussian noise is adopted as the background noise^39^ whose power spectrum density is determined by our integrated noise model.^26^ At frequencies below 5 kHz (confined by the 10 kHz sampling rate), the noise has four components: flicker noise, electrode noise, white thermal noise, and dielectric noise. Their respective importance increases successively from low to high frequencies. The related parameters are selected as the typical values of SiN_*x*_ nanopores from our previous measurements.^26^ The amplitude, reflecting the power, of the background noise can be tuned for data sets with different SNR (more details in Note 3 of the SI).

In addition, two kinds of variations of the baseline, *i*.*e*., sudden jumps and slow fluctuations, are introduced to represent the perturbation. The former generates randomly appearing steps in the baseline to mimic the temporal adsorption–desorption of some objects near and in the pore.^5,40^ The latter simulates the instability of the nanopore, which can be caused by the fluctuation of the pore membrane, dynamics of contamination, *etc*.^41,42^ It is described by the superposition of eight terms of sine and cosine functions. The probability of appearance of the perturbation steps, the amplitude of the steps, and the amplitude of the slow fluctuations are carefully tuned to approach the real common situations in experiments (more details in Note 3 of the SI). Involving the baseline variations can largely enhance the robustness of our neural network. Accustomed to complicated scenarios in the training process, the B-Net can handle real measured data more competently.

In signal generation, both diameter and thickness of a nanopore are fixed to 20 nm. Typical experimental conditions are adopted, *i*.*e*., a bias voltage of 300 mV, a 100 mM KCl electrolyte, and surface charge density of −0.02 C/m^2^. Three parameters in the signal generation program, *i*.*e*., concentration of the nanospheres (aforementioned *C*_np_), diameter of translocating nanospheres (aforementioned *D*_np_), and duration of translocation, are systematically varied for each data set since these three parameters have a direct correlation with the three most important features of the translocation signal, *i*.*e*., frequency, amplitude, and duration. In each data set, *C*_np_ is varied from 0.01 to 1 nM, changing in logarithmic scale (*i*.*e*., 20 different values in total), while *D*_np_ is varied from 3 to 17 nm, with a 1 nm step (*i*.*e*., 15 different values in total). The duration of translocation is directly assigned to 0.5, 1, 1.5, 3, and 5 ms (*i*.*e*., five different values in total). To evaluate the performance of the B-Net for different SNR conditions, SNR is varied from 0.25 to 4 (*i*.*e*., five different values in total). It is worth noting that SNR is defined as the ratio of spike amplitude to the peak-to-peak value of the background noise. The peak-to-peak value of the Gaussian noise is estimated to be six times of its root-mean-square (RMS) value,^5^ while the noise RMS can be calculated by the root square of the integration of the noise power spectrum density in the range of the bandwidth. All the data are generated using a homemade program implemented in MATLAB.

#### Experimental Data

In order to evaluate the performance of our algorithm on experimental data from the laboratory, two groups of translocation experiments are implemented in truncated pyramid nanopores (TPPs). The TPPs are formed in the single-crystal silicon layer of a silicon-on-insulator wafer. Their shape naturally adopts a truncated pyramid by exploiting the difference of wet etching rate of the ⟨100⟩ and ⟨111⟩ crystal orientations of single-crystal silicon. Details about the nanopore fabrication can be found in our previous work.^10^ Two different TPPs were used in our experiments, one with a side length of 7.5 nm and another 16 nm, both in a 55-nm-thick silicon layer, for DNA and protein *streptavidin* translocation, respectively.

λ-DNA and *streptavidin* were selected as two typical examples of translocating analytes, representing two typical examples of long and spherical objects, respectively. A λ-DNA strand has 48 502 nucleobase pairs in a double-stranded helix structure with a total length of about 16 μm.^43^ It carries a high density of negative charge, making its translocation usually governed by the electrophoretic force. *Streptavidin* is sphere-like with a diameter of 6 nm.^2^ It is weakly negatively charged in a pH neutral environment, and the translocation is usually dominated by the electroosmotic flow. They were purchased from Merck KGaA, Darmstadt, Germany, and used without further purification. The λ-DNA and *streptavidin* were dispersed in 500 mM KCl electrolyte with a concentration of 78 pM and 84 nM, respectively.

The nanopore chip was mounted on a custom-made poly(methyl methacrylate) (PMMA) flow cell and sealed using two polydimethylsiloxane (PDMS) O-rings (8 mm in inner diameter) on the two sides.^10^ Two compartments both filled with KCl solution were separated by the chip, and the only path of ionic current was through the nanopore. A pair of Ag/AgCl pseudoreference electrodes (2 mm in diameter, Warner Instruments, LLC) was used to apply a bias voltage and to collect the ionic current. Electrical measurements were controlled using a patch clamp amplifier (Axopatch 200B, Molecular Devices Inc.). During the translocation experiments, analyte dispersions were added to both compartments. The ionic current was converted to the digital signal by Axon Digidata 1550A (Molecular Devices LLC) and recorded by the software Axon pCLAMP 10 (Molecular Devices LLC). The λ-DNA translocation was measured at a 10 kHz sample rate with 2 kHz analog bandwidth, while the *streptavidin* translocation was detected at a 20 kHz sampling frequency with a 10 kHz bandwidth.

#### Traditional Data Processing Method

The recognition of translocation events by means of traditional data processing methods is based on the amplitude of spikes. Here, we use a homemade MATLAB program to locate the translocation spikes in current traces and extract the three parameters: translocation frequency, amplitude, and duration. In the program, the function *findpeaks* is adopted with the *MinPeakProminence* method. An amplitude threshold is defined by the user regarding the RMS of the background noise level. In the following discussion, this threshold is tuned from 4 to 25 multiples of the background noise RMS to demonstrate the dependence of the results on the threshold selection.

#### Standard Database

A standard database for testing the performance of the translocation signal has been established.^44^ There are two categories of data, *i*.*e*., generated and experimental. For each data set of the generated data, a current trace without background noise is also offered, apart from its counterpart trace with noise. Furthermore, the spike information, *i*.*e*., amplitude, start time, end time, and duration, is given as the standard answer for corresponding traces. For the experimental data, the current traces are trimmed with the same length. In addition, all the other important information, such as simulation and experiment conditions, is offered. This database is completely open to the public and can be used to train, validate, and test different algorithms on the same page. The database will be enriched, updated, and maintained continually.

### B-Net Training Process Using Artificially Generated Train and Validation Data Sets

In order to train and validate the B-Net, the artificially generated data introduced in the section Data Preparation were used. Afterward, the B-Net was evaluated on both artificial and experimental data also introduced in the section Data Preparation. The traces in each data set were segmented in temporal windows of 0.5 s. In regard to the artificially generated data sets, we ended up with 60 000 temporal windows for training, 30 000 for validation and 30 000 for testing. Regarding experimental real data sets, we ended up with 852 temporal windows from our λ-DNA experimental data set and 1512 windows from our *streptavidin* experimental data set.

The B-Net is composed of two networks, ResNet 1 and ResNet 2, which were trained separately. Five different B-Net instances were trained using five different SNRs in the artificial data sets (SNR = 4, 2, 1, 0.5, and 0.25). In all cases, smooth *l*_1_-loss and SGD optimization were adopted. Smooth *l*_1_-loss can be seen as a blend of *l*_1_-loss and *l*_2_-loss. It returns the *l*_1_-loss when the absolute value of the argument is high and the *l*_2_-loss when such an argument is close to zero. In eq 1, the quadratic segment smooths the *l*_1_-loss near |*x* – *y*| = 0. This loss is less sensitive to outliers than other measures such as the mean-square error loss. In some cases, it could prevent exploding gradients, which is desirable in networks with architectures like the one used in this work.^45^
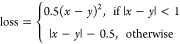
1Smooth *l*_1_-loss combines the advantages of *l*_1_-loss (producing steady gradients for large values of |*x*–*y*|) and *l*_2_-loss (producing fewer oscillations during updates when |*x* – *y*| is small).

For SNR equal to 4, 2, and 1, a batch size of 32 temporal windows and an initial learning rate of 0.001 were used. The learning rate was decreased by 90% every 10 epochs for ResNet 1 and every 20 epochs for ResNet 2. For SNR equal to 0.5 and 0.25, a batch size of 8 windows and an initial learning rate of 0.001 were used. The learning rate was decreased by 20% every 10 epochs for ResNet 1 and every 20 epochs for ResNet 2. During the ResNet 2 training procedure, temporal windows without translocation events were ignored. To predict average translocation amplitude and duration, ResNet 2 always received windows containing at least one translocation. On the other hand, ResNet 1 processed all temporal windows during training with and without translocation events. During training, ResNet 2 did not receive the prediction of the number of pulses in the window from ResNet 1; it instead received such information from the ground truth in the data set. Such information was injected in ResNet 2 in its FFNN.

Validation after each epoch in the training process was implemented on each network. For ResNet 2, the network with the minimum relative errors of amplitude and duration won and was saved in each epoch iteration. The relative error is defined as
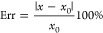
2where *x* is the predicted value and *x*_0_ the true value. ResNet 1, on the other hand, may process temporal windows without translocation spikes. Consequently, it has a division-by-zero risk if the relative error in eq 2 is applied. Therefore, for the ResNet 1 validation, relative percent difference (RPD), defined by eq 3, was adopted to represent the relative error of the predicted pulse number.
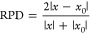
3

### Evaluation (Testing) Process Using Artificially Generated Test Data Set

In order to evaluate the B-Net, a held-out artificially generated data set, used for neither training nor validation, was used. This data set has the same size as the validation data set. Since it is artificially generated, it is completely labeled. Therefore, for each temporal window from the traces, the ground-truth features were known during data generation, including the real number of pulses and the real average amplitude and duration of the pulses in the temporal windows. Then, the relative error between the labeled signals and the predictions produced by the B-Net was calculated, following eq 2.

Unlike training and validation, during evaluation, the B-Net works as follows. First, ResNet 1 processes the temporal window and outputs an estimation of the number of pulses in the window. If the number of pulses predicted by ResNet 1 is 0, ResNet 2 will not process the input and the B-Net will predict 0 for pulses, 0 average amplitude, and 0 average duration. On the other hand, if ResNet 1 predicts one or more pulses in the window, then ResNet 2 will process the input and predict two aforementioned features, *i*.*e*., the average amplitude and duration of the pulses in the window. It is important to highlight that ResNet 2 was trained using only temporal windows with one or more translocation events (pulses). It never received windows without pulses, consequently, and only in the case of correct prediction of number of pulses did ResNet 1 prevent ResNet 2 from processing windows without pulses, for which it was not trained.

In addition, considering the division-by-zero risk, those two rules below were followed for the evaluation stage:

- If ResNet 1 correctly predicts 0 pulses in a window, then we consider 0% error for all features, *i*.*e*., number of pulses, average amplitude, and average duration.
- If ResNet 1 erroneously predicts more than 0 pulses in a window, when the ground truth is actually 0, then we consider 100% error for all features, *i*.*e*., number of pulses, average amplitude, and average duration.

## Supporting Information Available

Signal processing flow for nanopore sensing; network architecture; physical models and data generation; evaluation (testing) results of an artificially generated test data set for different SNRs; training history using artificially generated train and validation data sets for different SNRs; comparison between the results from our neural network and the traditional algorithm; translocation features of λ-DNA and *streptavidin* extracted by the B-Net; translocation frequency of *streptavidin* (PDF)

## Notes

The authors state that this manuscript has be published as a preprint in arXiv: Dematties, D.; Wen, C.; Pérez, M. D.; Zhou, D.; Zhang, S.-L. Deep Learning of Nanopore Sensing Signals Using a Bi-Path Network. **2021**, 2105.03660. *arXiv*. https://arxiv.org/abs/2105.03660 (accessed May 8, 2021).

## Notes

The generated data sets for all the combinations of the parameters and the experimental detests of λ-DNA and *streptavidin* translocation are available at https://zenodo.org/record/5013856#.YNYAxvkzZPY. The code for B-Net is available at https://github.com/dariodematties/NANOPORE_TRANSLOCATIONS and https://zenodo.org/record/5031817#.YNYA5_kzZPY.
