# Supplementary material for: Deep Learning of Nanopore Sensing Signals Using a Bi-Path Network
Source: ACS Nano. 2021 Aug 17;15(9):14419–29. doi: 10.1021/acsnano.1c03842 (PMC8482760; doi:10.1021/acsnano.1c03842)
Supplement: Supplementary file 1 — nn1c03842_si_001.pdf [file nn1c03842_si_001.pdf]

Supporting Information

Deep Learning of Nanopore Sensing Signals Using a Bi-Path Network

*Dario Dematties,[1]‡ Chenyu Wen,[2]‡ Mauricio David Pérez,[2] Dian Zhou,[3] Shi-Li Zhang[2]**

[1]Instituto de Ciencias Humanas, Sociales y Ambientales CONICET Mendoza Technological Scientific Center, Mendoza M5500, Argentina

[2]Division of Solid-State Electronics, Department of Electrical Engineering, Uppsala University, SE-751 03 Uppsala, Sweden

[3]Department of Electrical and Computer Engineering, University of Texas at Dallas, Richardson, TX 75080, USA

* To whom correspondence should be addressed: shili.zhang@angstrom.uu.se.

‡These authors contributed equally.

Table of Contents

Note 1. Signal processing flow for nanopore sensing

Note 2. Network architecture

Note 3. Physical models and data generation

Resistance model and open-pore current

Translocation model and translocation spikes

Noise model and background noise generation

Baseline variation

Note 4. Bi-path Network (B-Net) evaluation (testing) results when processing artificially generated test dataset for different Signal-to-Noise Ratio (SNR)

Note 5. B-Net training history using artificially generated train and validation datasets for different SNR

Note 6. Comparison between the results from our neural network and the traditional algorithm

Artificially generated dataset, varying diameter of nanopore (D_{np})

Artificially generated dataset, varying concentration of nanospheres (C_{np})

Artificially generated dataset, varying duration

Note 7. Translocation features of λ-DNA and *streptavidin* extracted by the B-Net

Note 8. Translocation frequency of *streptavidin*

Note 1. General signal processing flow for nanopore sensors

The typical signal processing flow for nanopore sensors is summarized in Figure S1.

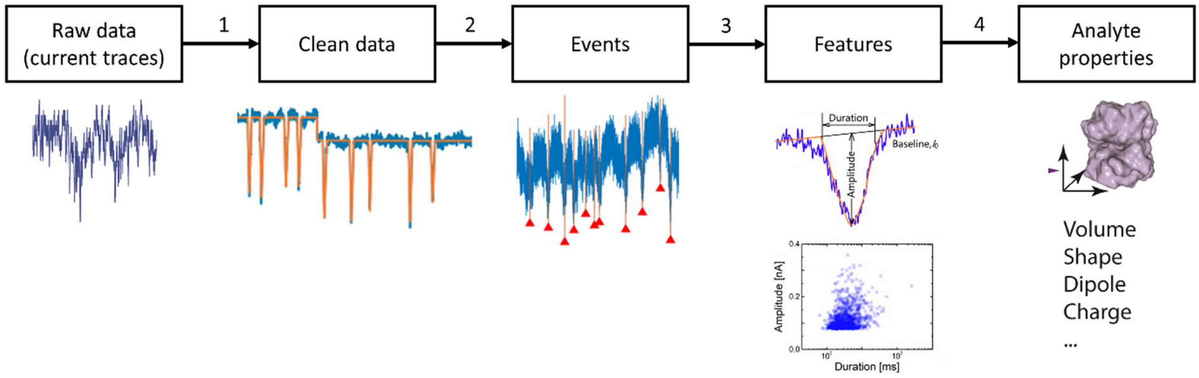

Figure S1. Data processing flow of the nanopore translocation signal.

• Step 1. Raw data is denoised to form clean data, which can be achieved using low pass filters in frequency domain.[1] In time domain, the baseline and blockage level can be traced and cleaned up from the background noise by averaging with a dynamically adjustable threshold, such as CUSUM algorithm.[2] Algorithms based on other theory, such as estimation theory, *e.g.*, Karman filter,[3] and wavelet transform[4] are also adopted.

• Step 2. Translocation events, represented as spikes, are recognized and extracted from the current traces. This procedure is usually based on a user-defined threshold of the amplitude as a criterion to separate a true translocation generated spike from a noise fluctuation.[5]

• Step 3. Features of these spikes are extracted based on physical models, such as ADEPT,[6] peak analysis algorithms, such as DBC,[7] and algorithms of feature analysis in frequency domain, such as Fourier transform and cepstrum.[8]

• Step 4. Properties of the translocating analytes are inferred from the extracted features. Based on simple physical models, the amplitude of spikes is correlated to the size and shape of analytes.[9,10] The duration is related to the translocation speed and nanopore-analyte interaction, reflecting the physiochemical properties, such as mass, charge, dipole, and hydrophobicity.[11,12] The frequency of spikes concerns the concentration of analytes.[12,13] Furthermore, details of translocation waveform are considered by more sophisticated models,[12,14] such as that using the fingerprint feature of blockage current distribution to distinguish 10 kinds of proteins.[15] In this step, Machine Learning (ML)-based classification algorithms are widely adopted to cluster the events and associate them to different analytes, such as support vector machine,[16] Convolutional Neural Network (CNN),[17] logistic classifier,[18] and decision tree.[19,20]

Note 2. Network architecture

Nowadays, thanks to the advent of the *Representation Learning* theory,[21] machines can automatically *discover representations*, which is relevant for the detection of specific features demanded by the network designer. In general, Deep Learning (DL) is known as a set of multi-layer representation-learning methods. Starting from the raw input, these networks transform the representations, one layer at a time by means of simple, but non-linear computations. Representations at higher layers are considered to be more acute or *pertinent* for the features pretended to be extracted from the network. DL has shown outstanding breakthroughs in the last years in areas ranging from Computer Vision (CV) to Natural Language Processing (NLP), from science to engineering, from medicine to material and computer sciences, *etc*.[21-23] By only individualizing the correct cost function in a subsystem at the top of the network, errors are then backpropagated after each iteration and the weights of the network are automatically adjusted to do better predictions in response to subsequent inputs in a training dataset.[22]

In this work, we introduce a network named B-Net. In the B-Net, a specific architecture called Residual Neural Network (ResNet) is adopted. ResNet is an artificial Deep Neural Network (DNN) that uses CNNs (A CNN is a highly utilized sub-type of DNN mostly applied to analyzing visual imagery) and skips connections, or shortcuts that jump over some layers.[24,25] The main motivation for skipping layers is to avoid the problem of *vanishing and exploding gradients*. By means of these *skip connections* the network can reuse activations from previous layers until posterior layers learn their weights. In the worst scenario, layer l+1 is able to receive an intact output from layer l-1 if layer l has not yet learned a proper weigh configuration. In this way, instead of making layer l+1 receive a potentially harmful representation from an *immature* layer l, it can avoid utilization of layer l and instead receive better information from previous layers in the network until a specific training point at which layer l eventually learns its correct weights. It is worth noting that l±1 is used here in a figurative way to favour a clearer explanation. In real implementations, skipping connections actually jump two or more layers and a more realistic scenario would describe something like l±2 or l±3. In this manner ResNet would never go *off rail* in terms of the correct manifold in its learning space. Nowadays, ResNet is considered a classic architecture usually employed as a backbone for many computer vision tasks. This architecture gained its privileged place among other highly effective DL architectures winning the ILSVRC 2015 in image classification, detection, and localization, as well as the MS COCO 2015 detection, and segmentation.[25]

In this work, we have used the ResNet18 architecture. This is one of the standard architectures utilized in DL frameworks such as Pytorch. PyTorch is an open-source ML library based on the Torch library. It is essentially developed by Facebook’s AI Research (FAIR) laboratory and is used for ML applications such as computer vision and natural language processing. Currently, ResNet 18 presents the performance with Top-1 and Top-5 error rates of 30.24% and 10.92% on image dataset, respectively.[24] As its name suggests, it has 18 layers in its standard version, 17 of which are convolutional while the last layer is a fully connected one. The first layer is an isolated one-dimensional convolution with an input of one one-dimensional channel, a kernel size of 3, a stride of 1, a padding of 1, without bias components and an output of 64 one-dimensional channels. Following this layer, there is a one-dimensional batch normalization layer for the 64 channels and a relu activation function. Then there are 4 groups of 2 basic residual blocks. Each basic residual block is composed of 2 one-dimensional convolutions (the residual part) and a skip connection that bypasses both convolutional layers. Each one-

dimensional convolution in the basic residual block has a kernel of size 3, a stride of 1 and a padding of 1. Each convolutional layer is followed by a group normalization layer, unlike the original architecture in which batch normalization is used. This arrangement improves performance significantly given the reduced batch sizes managed during training. There is an instance of a relu activation function between both convolutions plus group normalizations. As stated above, the network has 4 groups of 2 basic residual blocks; the blocks in the first group have 64 channels in their outputs, the blocks in the second group have 128 channels, the blocks in the third group have 256 channels, and the blocks in the last group have 512 channels. Afterwards, information is average-pulled, the tensors are flattened, and finally such flattened tensors are processed by two fully connected layers, instead of one layer as in the original architecture. We have used two linear layers instead of one at the end in order to compensate for any information scarcity that could be produced in the processing of one-dimensional data.

Note 3. Physical models and data generation

Resistance model and open-pore current

To determine the baseline of the current trace, *i.e.* open-pore current, our previously established resistance model based on the concept of effective transport length is adopted here.[26] The resistance of a cylindrical nanopore can be expressed as

$$R = \frac{4\rho L_{eff}}{4\pi d_p^2} \tag{1}$$

where, ρ is the resistivity of the electrolyte and d_p the diameter of the nanopore and L_{eff} the effective transport length of the nanopore that is defined as the sum of the distances from the location inside the nanopore where the electric filed is the highest to the two opposite points along the central axis of the pore where the electric fields both fall to e^{-1} of the maximum. For a cylinder pore, L_{eff} is[26]

$$L_{eff} = 0.92 d_p + h \tag{2}$$

where, h is the thickness of nanopore. The resistivity of the electrolyte is determined by its salt concentration, c_0 and the mobility of cations, μ_c, and anions, μ_a.

$$\rho = (q N_A c_0 (\mu_c + \mu_a))^{-1} \tag{3}$$

where, q is the element charge and N_A the Avogadro constant. The surface charge on the nanopore sidewall also contributes to the conductance. The surface conductance can be expressed as

$$G_s = \mu\sigma \frac{\pi d_p}{h} \tag{4}$$

where, μ is the mobility of the counterions in the surface electric double layer and σ the surface charge density. Thus, at a given bias voltage V, the open-pore current is

$$I_0 = V(G_s + 1/R) \tag{5}$$

Values of the parameters used in this model are listed in Table 1.

Table 1. Parameters used in the resistance model

Parameter	Unit	Value	Reference
q	C	1.6×10^{-19}	
N_A	mol^{-1}	6.02×10^{23}	
c_0	mM	100	
d_p	nm	20	
h	nm	20	
V	mV	300	
σ	Cm^{-2}	−0.02	(27, 28, 29)
μ_c	$m^2V^{-1}s^{-1}$	7.575×10^{-9}	(30)
μ_a	$m^2V^{-1}s^{-1}$	7.874×10^{-9}	(30)

Translocation model and translocation spikes

The amplitude of the translocation spikes is simply determined by a steric blockage model[31] that concerns the ratio of the cross-section area of the nanopore to that of the translocating nanosphere.

$$\Delta I = I_0 \frac{D_{np}^2}{d_p^2} \tag{6}$$

where, D_{np} is the diameter of the translocating nanosphere. The shape of translocation spikes is approximated by a triangle, as shown in Figure S2. In a spike, the current decreases from the open-pore current I_0 in the first 40% of the duration time to reach the minimum I_b, and then increases back to I_0 in the rest of the 60% duration.

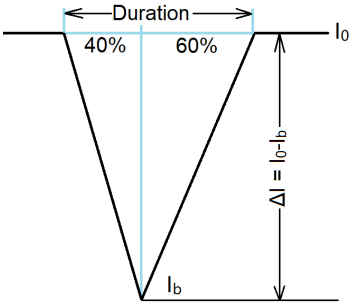

Figure S2. The triangular spike waveform used in signal generation.

The probability of finding a spike at certain time point is set to be proportional to the nanosphere concentration C_{np} and exponentially dependent on bias voltage.

$$P = k_0 C_{np} e^{\frac{qV}{k_B T}} \tag{7}$$

where, k_0 is a coefficient, k_B the Boltzmann constant and T temperature in Kelvin. During the signal generation, a time step δt of 0.1 ms, *i.e.* sampling rate of 10 kHz, is specified. Then, a state of either "open-pore" (o) or "blockage" (b) is randomly generated for each δt according to

a two-point distribution based on probability *P*. Each pore is sequentially accessed during one δt and I_0 is assigned for pores in state "o" and I_b for pores in state "b". It is worth to mention that a pore cannot change to state "o" until lasting for a certain duration time, once it is in state "b". The same algorithm of signal generation and the same dependence of spike appearance probability on analyte concentration and bias voltage have been adopted in our previous work for multiple nanopores.[32] Values of the parameters used in this model are listed in Table 2.

Table 2. Parameters used for translocation spike generation

Parameter	Unit	Value	Reference
k_0	nM^{-1}	9.1×10^{-8}	(32)
k_B	JK^{-1}	1.38×10^{-23}	
T	K	300	
C_{np}	nM	0.01−1	(32)
Duration	ms	0.5−5	

Noise model and background noise generation

A comprehensive noise model of solid-state nanopores has been established based on experimental characterization of SiN_x nanopores.[33] There are four distinct noise sources in the frequency range below 5 kHz: flicker noise S_{IF}, electrode noise S_{IE}, white thermal noise S_{IT}, and dielectric noise S_{ID}. The Power Spectrum Density (PSD) of these noise components are:

$$S_{IF} = \frac{\alpha_H I_0^2}{N_c f^{\beta 1}} \tag{8}$$

$$S_{IE} = \frac{\alpha_e}{f^{\beta 2}} \tag{9}$$

$$S_{IT} = 4k_B T / R \tag{10}$$

$$S_{ID} = 8\pi k_B T d_L C_{chip} f \tag{11}$$

with

$$N_c = 0.5\pi d_p^2 h c_0 N_A \tag{12}$$

where, α_H is a constant named Hooge's parameter, N_c the total number of conducting carriers in the nanopore, f the frequency, α_e the current noise parameter for the electrodes, β_i the factor for frequency dependency, d_L dielectric loss factor of the nanopore membrane and C_{chip} the parasitic capacitance of the membrane. The total PSD of the background current noise is the sum of these four components.

$$S_I = S_{IF} + S_{IE} + S_{IT} + S_{ID} \tag{13}$$

The time sequence of noise with the required length in time domain can be generated based on the white Gaussian noise generator in the MATLAB function library. Using the Fast Fourier Transform and Inverse Fourier Transform, the white Gaussian noise source can be modulated by the total PSD to become the colored Gaussian noise. Details of the algorithm can be found in the literature.[31] The amplitude of the noise can be tuned by a factor, so does SNR. Values of

the parameters used in the noise model are listed in Table 3.

Table 3. Parameters used in the noise model

Parameter	Unit	Value	Reference
α_H	1	1.9×10^{-4}	(34, 35)
β_1	1	1	(33)
β_2	1	1.5	(33)
α_e	A^2	2×10^{-24}	(33,36)
d_L	1	0.27	(33)
C_{chip}	nF	52	(33)

Baseline variation

Two kinds of baseline variations are involved in the signal generation: sudden jump of the baseline and slow fluctuation. The sudden jump of the baseline is achieved by randomly appeared steps on the baseline. The height of these steps is set to be 30% of ΔI with a random fluctuation of 10%. The number of steps appeared in a 10 s period is also a randomly generated number with an expectation of 30. The slow fluctuation of baseline is represented by the superposition of 8 terms of sine and cosine functions.

$$I_{fluc}=a_0I_0[a_1sin(\omega t)+a_2sin(2\omega t)+a_3sin(3\omega t)+a_4sin(4\omega t)+b_1cos(\omega t)+b_2cos(2\omega t)+b_3cos(3\omega t)+b_4cos(4\omega t)] \quad (14)$$

The general amplitude of the slow fluctuation is controlled by the factor a_0, which is 0.003 in the signal generation. a_i and b_i are amplitude coefficients. They are random numbers with an expectation of zero and Standard Deviation (STD) showing in Table 4.

Table 4. Parameters used in the baseline variations

Parameter	Fluctuation	Parameter	Fluctuation
a_1	0.5	a_2	0.5
a_3	0.1	a_4	0.05
b_1	0.5	b_2	0.5
b_3	0.1	b_4	0.05

Note 4. B-Net evaluation (testing) results when processing artificially generated test dataset for different SNR

In Figures S3, S5, S7, S9, and S11, we can see bathes each with 5 random temporal windows and how the corresponding B-Net instances predict the ground truth features in the windows. Likewise, we show the statistical results of B-Net in Figures S4, S6, S8, S10, and S12 when processing the artificially generated test dataset.

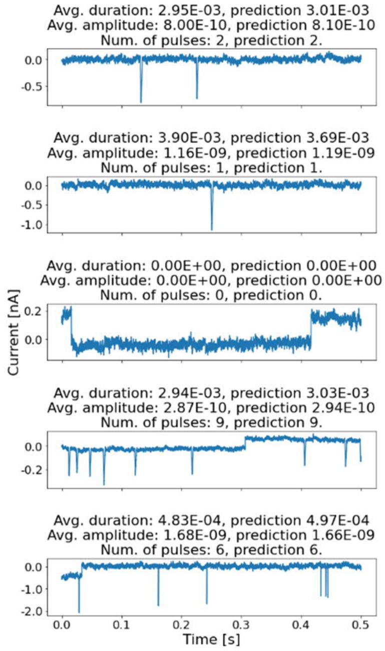

Figure S3. Examples of how the B-Net predicts features from temporal windows with SNR=4. In this batch with temporal windows, the average translocation duration error is 2.6%, average translocation amplitude error is 1.5%, average translocation counter error is 0.0% and 0 improper measures. Improper measures are produced when the ground-truth establishes 0 number of pulses but the network predicts one or more pulses.

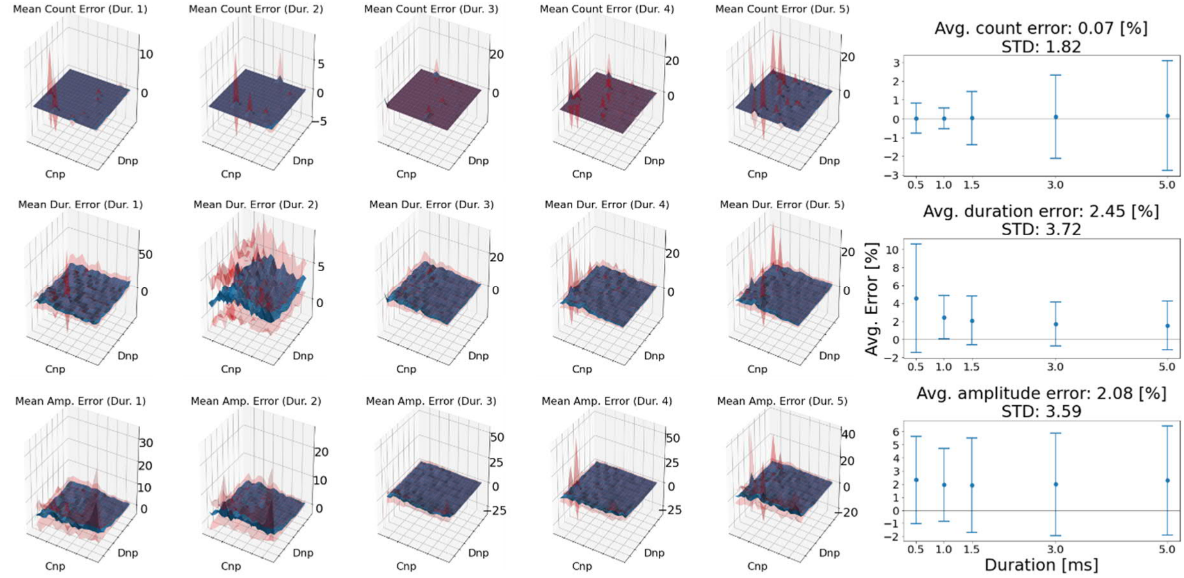

Figure S4. B-Net validation the SNR=4 dataset. (a) Surfaces of average prediction errors for the different features on temporal windows for different durations. Each surface is distributed through the space of C_{np} and D_{np} values. (b) Average prediction errors for each duration. Error bars correspond to STDs.

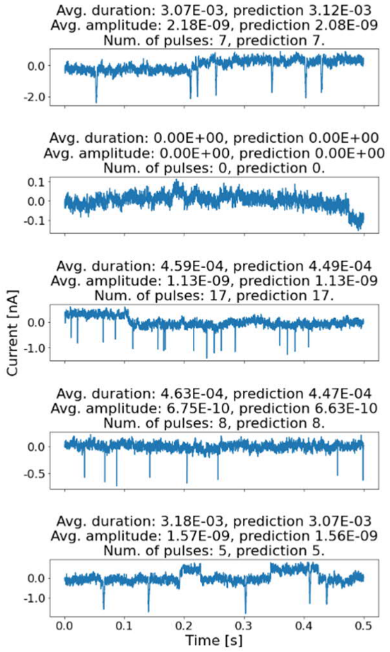

Figure S5. Examples of how the B-Net predicts features from temporal windows with SNR=2. In this batch with 5 temporals, the average translocation duration error is 2.1%, average translocation amplitude error is 1.4%, average translocation counter error is 0.0% and 0 improper measures.

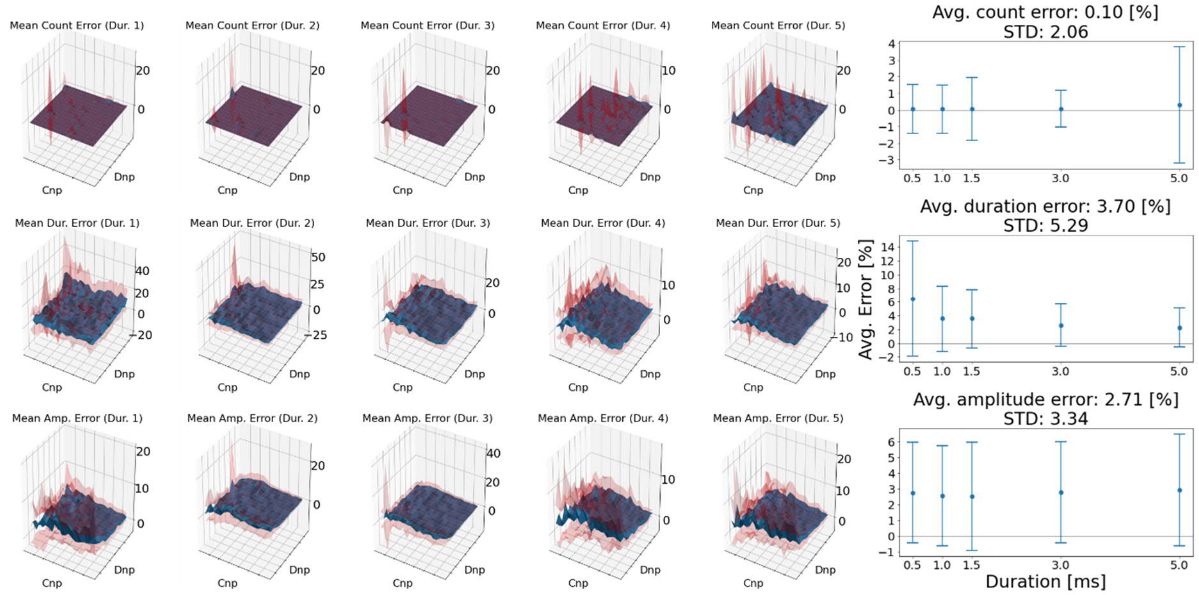

Figure S6. B-Net validation the SNR=2 dataset. (a) Surfaces of average prediction errors for the different features on temporal windows for different durations. Each surface is distributed through the space of C_{np} and D_{np} values. (b) Average prediction errors for each duration. Error bars correspond to STDs.

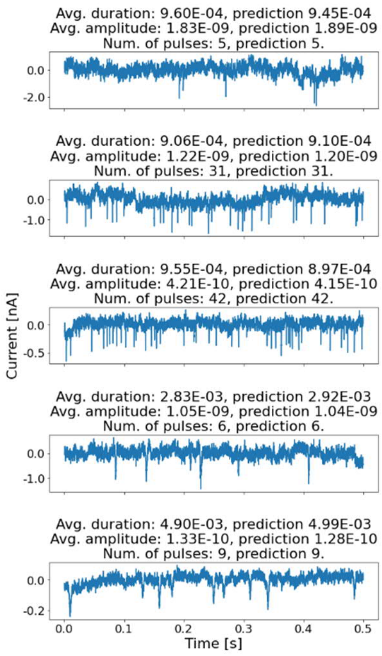

Figure S7. Examples of how the B-Net predicts features from temporal windows with SNR=1. In this batch with 5 temporal windows, the average translocation duration error: 2.6%, average translocation amplitude error: 2.2%, average translocation counter error: 0.0% and 0 improper measures.

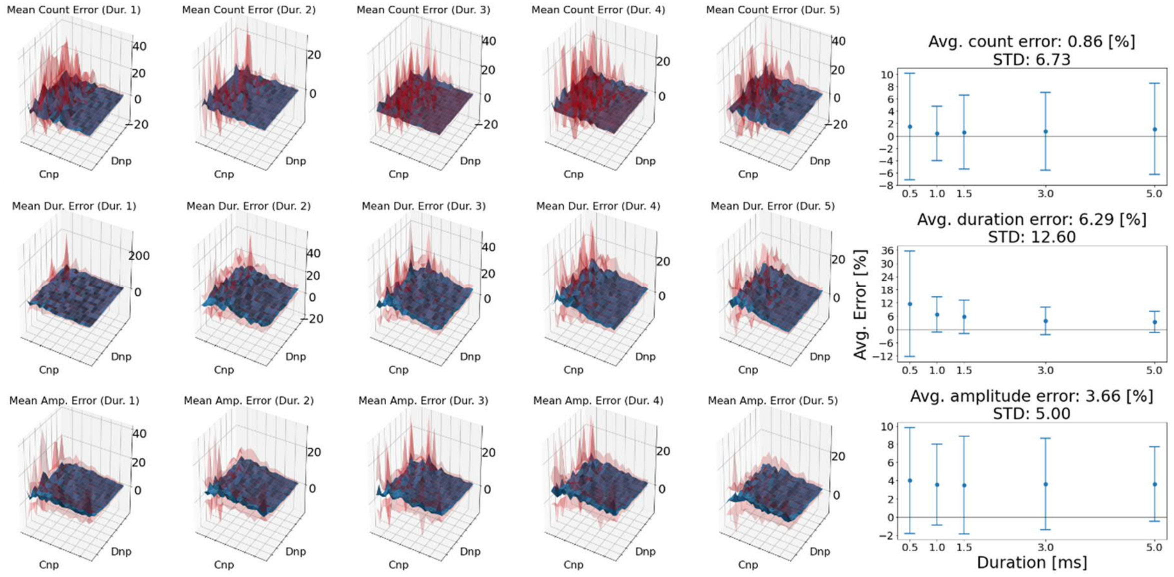

Figure S8. B-Net validation the SNR=1 dataset. (a) Surfaces of average prediction errors for the different features on temporal windows for different durations. Each surface is distributed through the space of C_{np} and D_{np} values. (b) Average prediction errors for each duration. Error bars correspond to STDs.

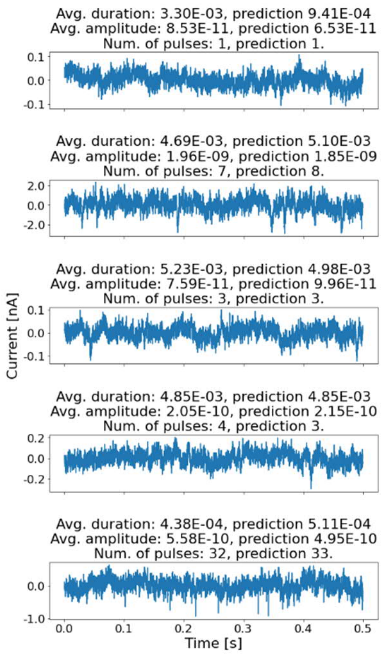

Figure S9. Examples of how the B-Net predicts features from temporal windows with SNR=0.5. In this batch with 5 temporal windows, the average translocation duration error: 20.4%, average translocation amplitude error: 15.2%, average translocation counter error: 8.5% and 0 improper measures

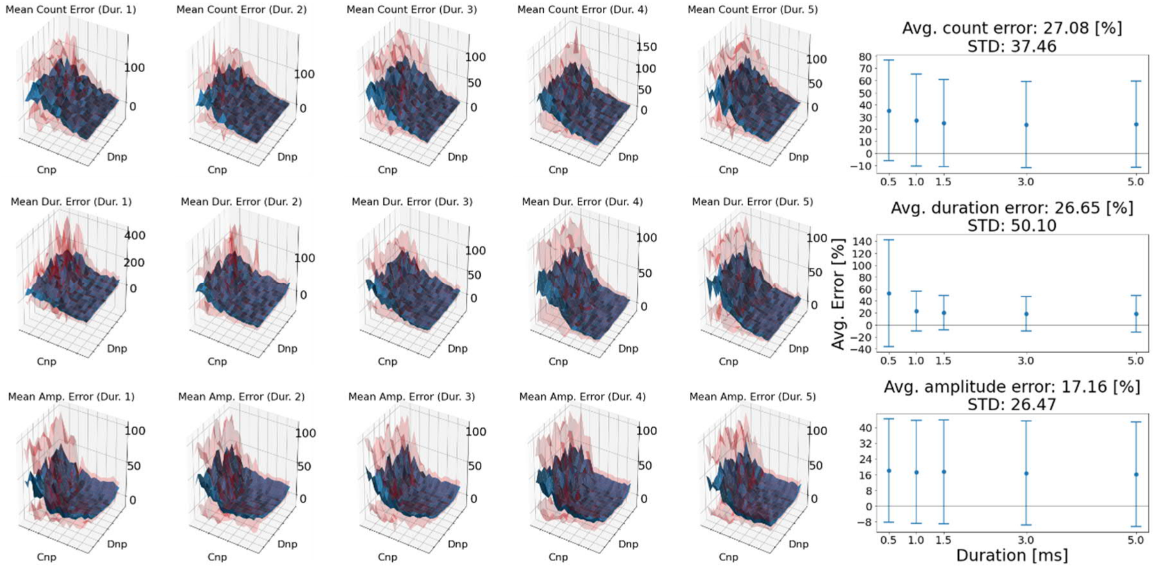

Figure S10. B-Net validation the SNR=0.5 dataset. (a) Surfaces of average prediction errors for the different features on temporal windows for different durations. Each surface is distributed through the space of C_{np} and D_{np} values. (b) Average prediction errors for each duration. Error bars correspond to STDs.

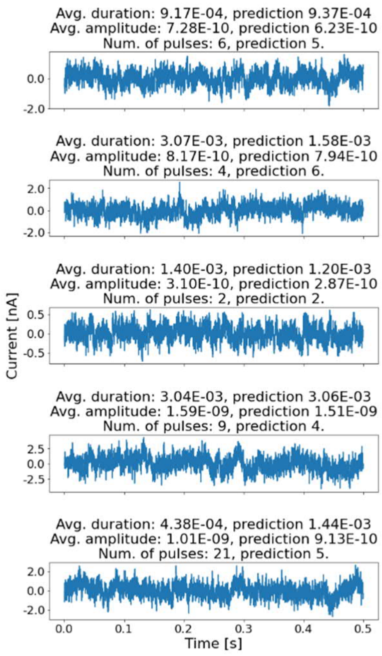

Figure S11. Examples of how the B-Net predicts features from temporal windows with SNR=0.25. In this batch of 5 temporal windows, the average translocation duration error: 58.8%, average translocation amplitude error: 7.9%, average translocation counter error: 39.7% and we find 0 improper measure.

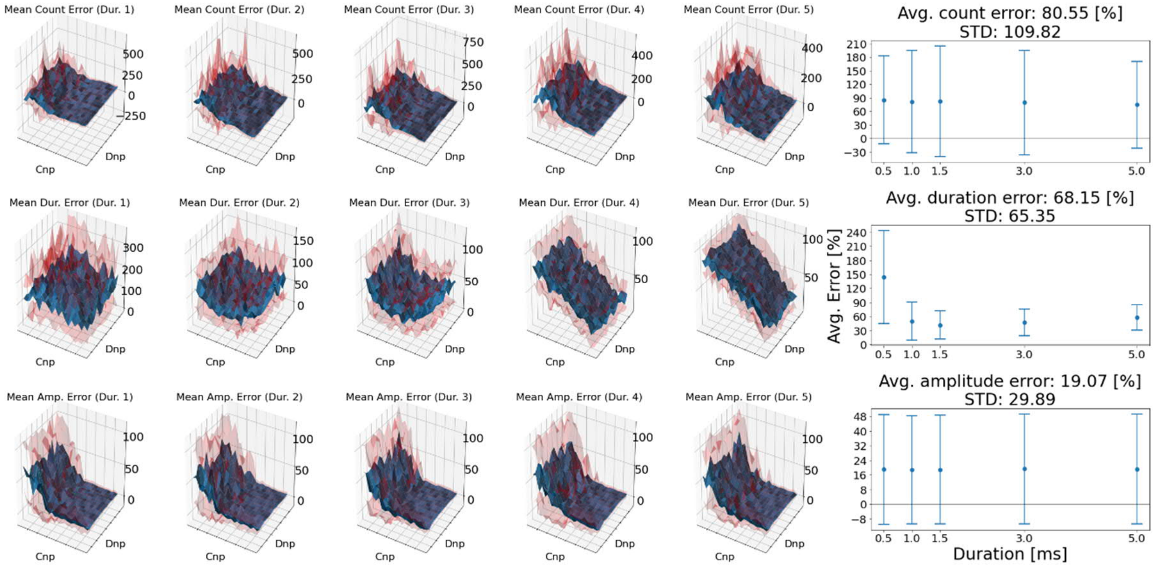

Figure S12. B-Net validation the SNR=0.25 dataset. (a) Surfaces of average prediction errors for the different features on temporal windows for different durations. Each surface is distributed through the space of C_{np} and D_{np} values. (b) Average prediction errors for each duration. Error bars correspond to STDs.

DL has shown advances in noise reduction in general, such as in image noise reduction[37-40] and audio signals, for speech enhancement[41, 42] and its application to cochlear implants.[43] From an architectural point of view, it is claimed that ResNets perform better than other simpler architectures which does not have skip connections. This hypothesis is based on experiments that suggest that ResNets have better noise stability, which is empirically supported for both simplified and fully-fledged ResNet variations.[44]

The relative errors stay near 1% when SNR > 2, while they can go straight up to 100% with SNR reaching 0.25. For a signal with SNR > 2, the neural network can almost thoroughly recognize every spike from the background noise. Increasing SNR does not influence the errors, indicating that these errors come from the system itself, *i.e.*, the neural network. When SNR is smaller than 1, the errors show a strong dependence on SNR, indicating that the interference on the spike recognition is from the background noise. It is almost impossible for traditional algorithms to correctly recognize translocation spikes in a background noise that has a similar amplitude to the spikes, *i.e.*, SNR=1.

Note 5. B-Net training history using artificially generated train and validation datasets for different SNR

In Figures S13, S14, S15, S16, and S17, we can see the training and validation history for each B-Net instance trained for this work. Among all the instances, we have each instance trained for a different level of noise (SNR=4, 2, 1, 0.5 and 0.25).

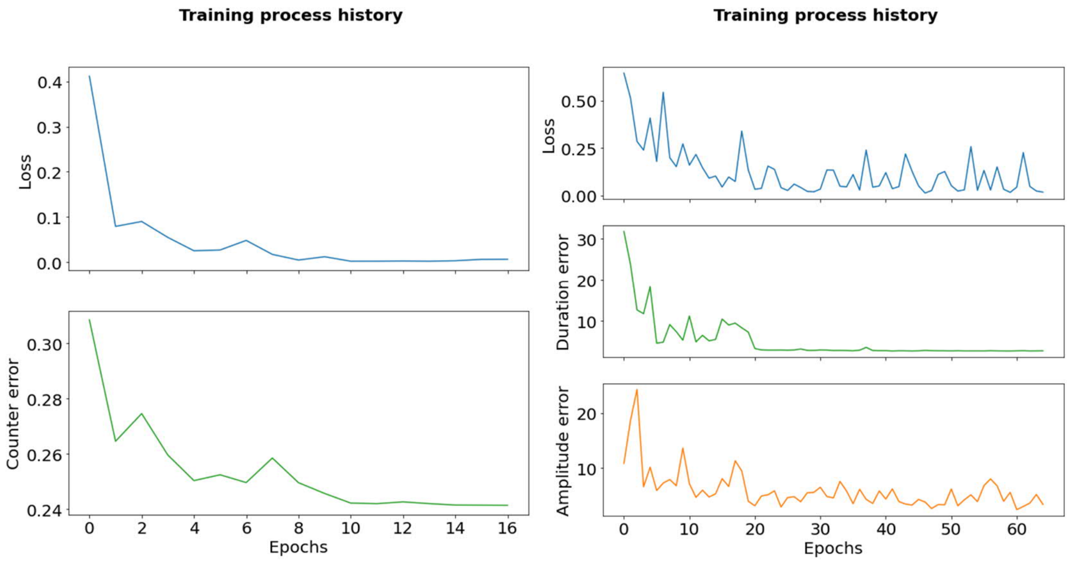

Figure S13. Training History trained on the SNR=4 dataset. ResNet 1. The total training time was 3 hours, 38 minutes and 50 seconds, while the average time during one epoch of training was 0 hours, 12 minutes and 52 seconds. Total training took 16 epochs. The best model was saved in epoch 16. (b) ResNet 2. The total training time was 15 hours 3 minutes and 47 seconds, while the average time during one epoch of training was 0 hours 13 minutes and 54 seconds. Total training took 64 epochs. The best model was saved in epoch 60 after 14 hours 10 minutes and 30 seconds.

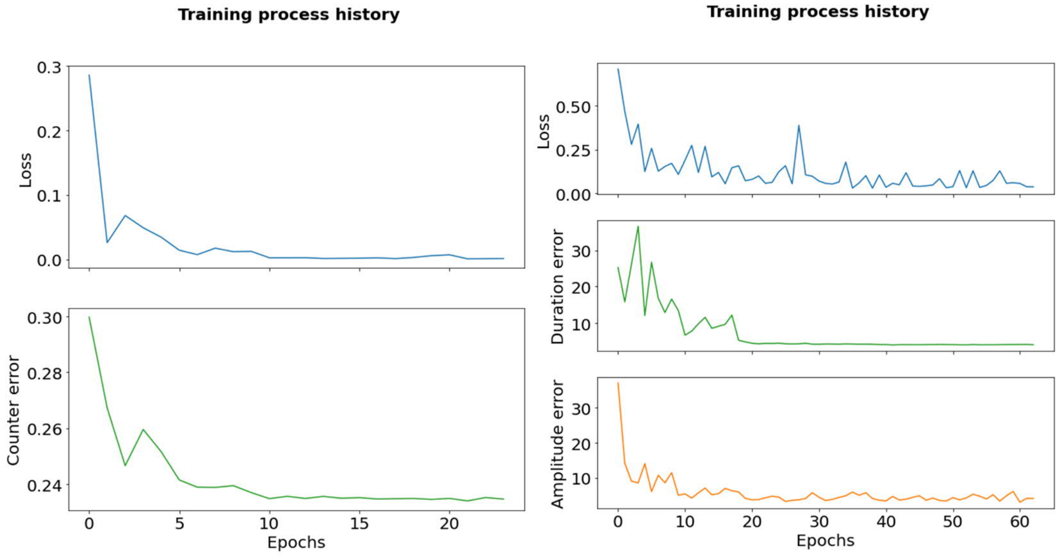

Figure S14. Training History trained on the SNR=2 dataset. (a) ResNet 1. The total training time was 5 hours 35 minutes and 55 seconds, while the average time during one epoch of training was 0 hours 13 minutes and 59 seconds. Total training took 23 epochs. The best model was saved in epoch 21 after 4 hours 55 minutes and 6 seconds. (b) ResNet 2. The total training

time was 16 hours 41 minutes and 36 seconds, while the average time during one epoch of training was 0 hours 15 minutes and 53 seconds. Total training took 62 epochs. The best model was saved in epoch 60 after 16 hours 13 minutes and 42 seconds.

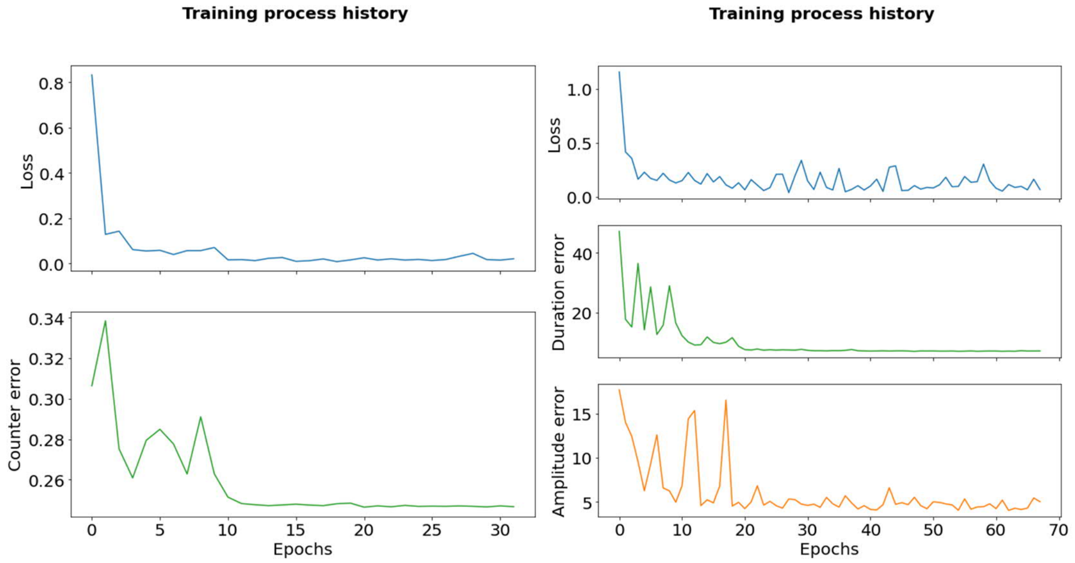

Figure S15. Training History trained on the SNR=1 dataset. (a) ResNet 1. The total training time was 7 hours 24 minutes and 29 seconds, while the average time during one epoch of training was 0 hours 13 minutes and 53 seconds. Total training took 31 epochs. The best model was saved in epoch 20 after 4 hours 45 minutes and 16 seconds. (b) ResNet 2. The total training time was 16 hours 35 minutes and 50 seconds, while the average time during one epoch of training was 0 hours 14 minutes and 38 seconds. Total training took 67 epochs. The best model was saved in epoch 54 after 13 hours 27 minutes and 39 seconds.

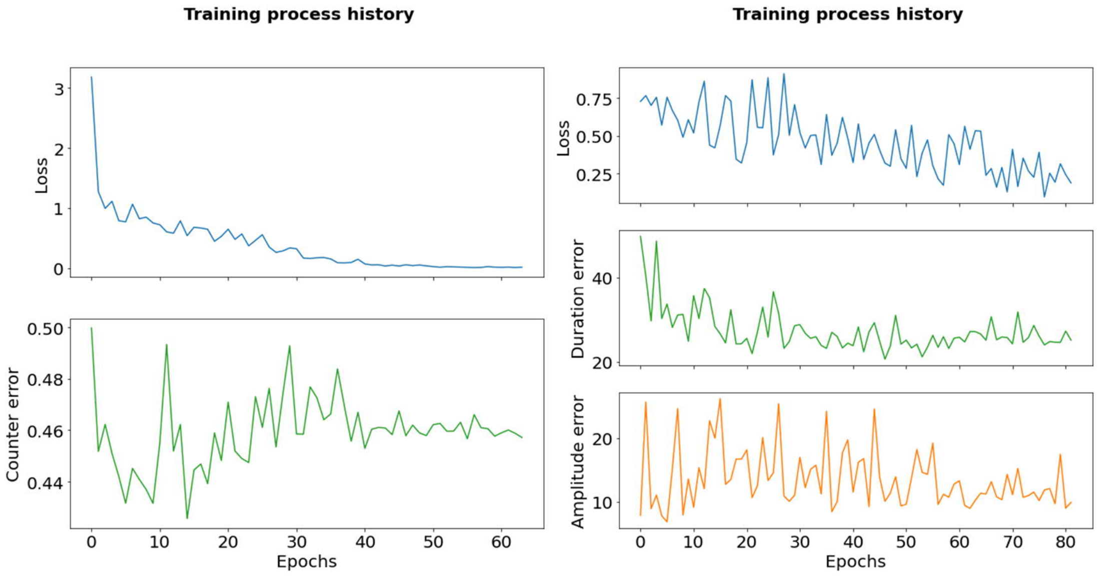

Figure S16. Training History trained on the SNR=0.5 dataset. (a) ResNet 1. The total training time was 16 hours 44 minutes and 52 seconds, while the average time during one epoch of training was 0 hours 15 minutes and 42 seconds. Total training took 63 epochs. The best model was saved in epoch 14 after 3 hours 54 minutes and 57 seconds. (b) ResNet 2. The total training time was 22 hours 35 minutes and 16 seconds, while the average time during one epoch of training was 0 hours 16 minutes and 31 seconds. Total training took 81 epochs. The best model was saved in epoch 46 after 12 hours 55 minutes and 28 seconds.

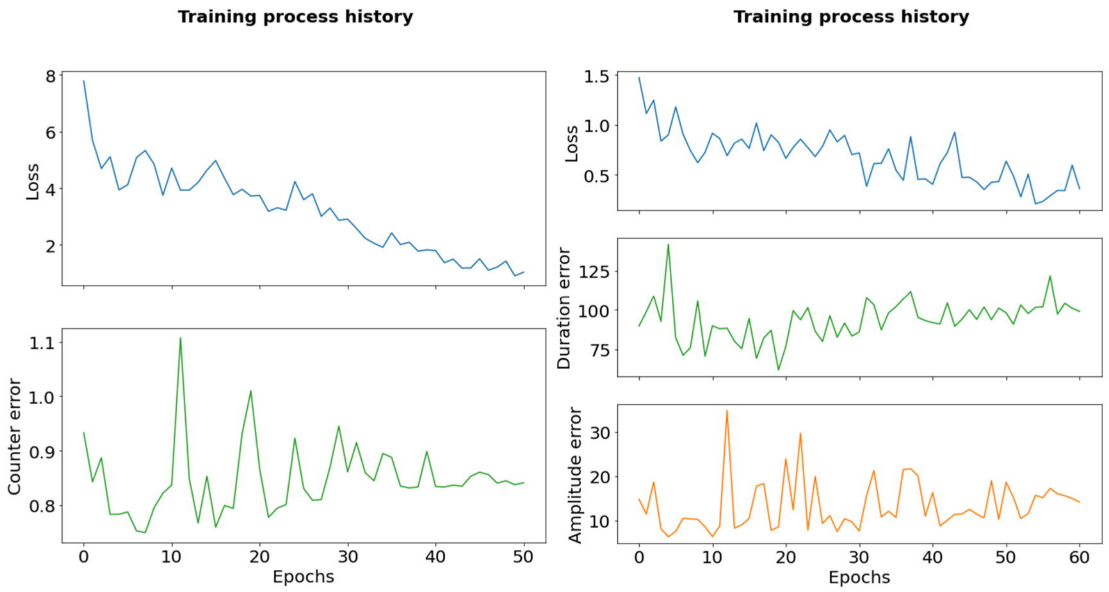

Figure S17. Training History trained on the SNR=0.25 dataset. (a) ResNet 1. The total training time was 13 hours 21 minutes and 16 seconds, while the average time during one epoch of training was 0 hours 15 minutes and 42 seconds. Total training took 50 epochs. The best model was saved in epoch 8 after 2 hours 9 minutes and 19 seconds. (b) ResNet 2. The total training time was 15 hours 53 minutes and 1 seconds, while the average time during one epoch of training was 0 hours 15 minutes and 37 seconds. Total training took 60 epochs. The best model was saved in epoch 19 after 5 hours 21 minutes and 12 seconds.

Note 6. Comparison between the results from our neural network and the traditional Algorithm

Enlarging the diameter of translocating nanospheres increases the amplitude (Figure S18a), while increasing the concentration of the nanospheres tends to raise the translocation frequency (Figure S19b). The extracted duration agrees well with the set value in the generated signal (Figure S20c). However, the average values of these features resulting from the traditional algorithm are dependent on the selection of threshold amplitude, as shown by the deviations among the dot-on lines in the respective figures. The number *n* after *th* (for threshold) in the legend denotes a specific threshold level, measured by the number of multiples of the peak-to-peak value of the background noise.

The prediction errors for the different features for both algorithms are compared in d-f of Figures S18-S20. The errors of the B-Net for frequency estimation are almost zero in comparison to the traditional algorithm. This can be seen in e of Figures S18-S20, where errors are four orders of magnitude below what the traditional algorithm produces for several D_{np}, C_{np} and duration values. The error of the B-Net is up to three orders of magnitude lower in d of Figures S18-S20 and up to two orders of magnitude below in f of Figures S18-S20. In addition, the relative errors of the feature extraction for the traditional algorithm are also highly dependent on the selection of amplitude threshold, indicating the subjectivity of the algorithm as well.

Artificially generated dataset, varying D_{np}

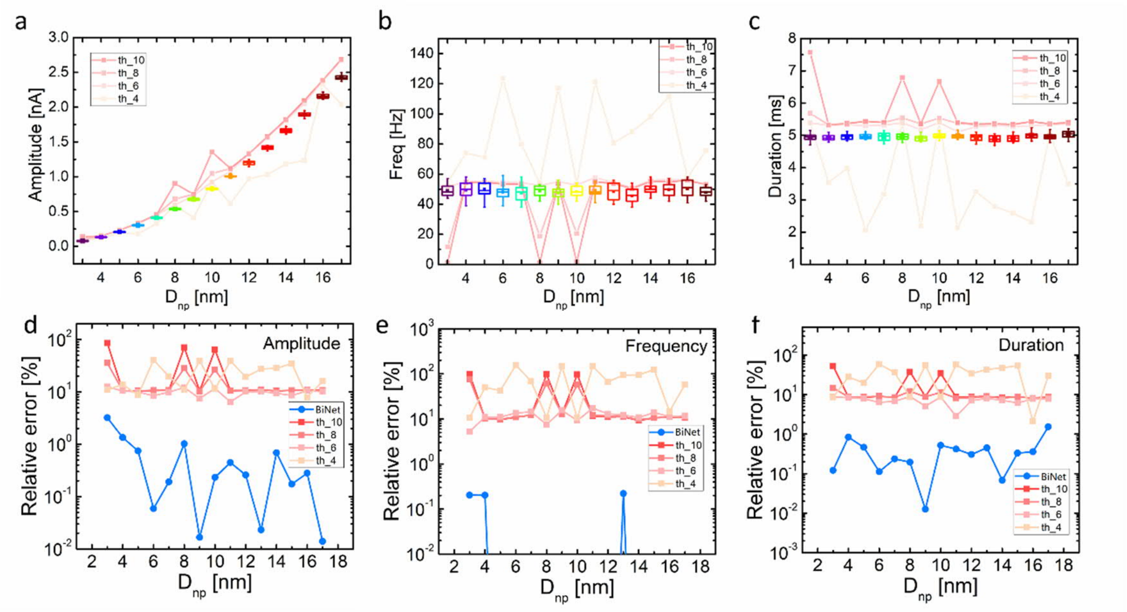

Figure S18. Comparison of the results from our neural network B-Net and those from the traditional algorithm on the dataset of varying D_{np}. (a-c) Box charts of the spike amplitude, frequency and duration for translocating nanospheres for different D_{np}, respectively, extracted by the B-Net. The average values of the spike amplitude, frequency and duration extracted by the traditional algorithm with different thresholds are shown by the dot-on lines in the corresponding figures for comparison. The relative errors of the spike amplitude, frequency and duration from the B-Net and the traditional algorithm are compared in d-f, respectively.

Artificially generated dataset, varying C_{np}

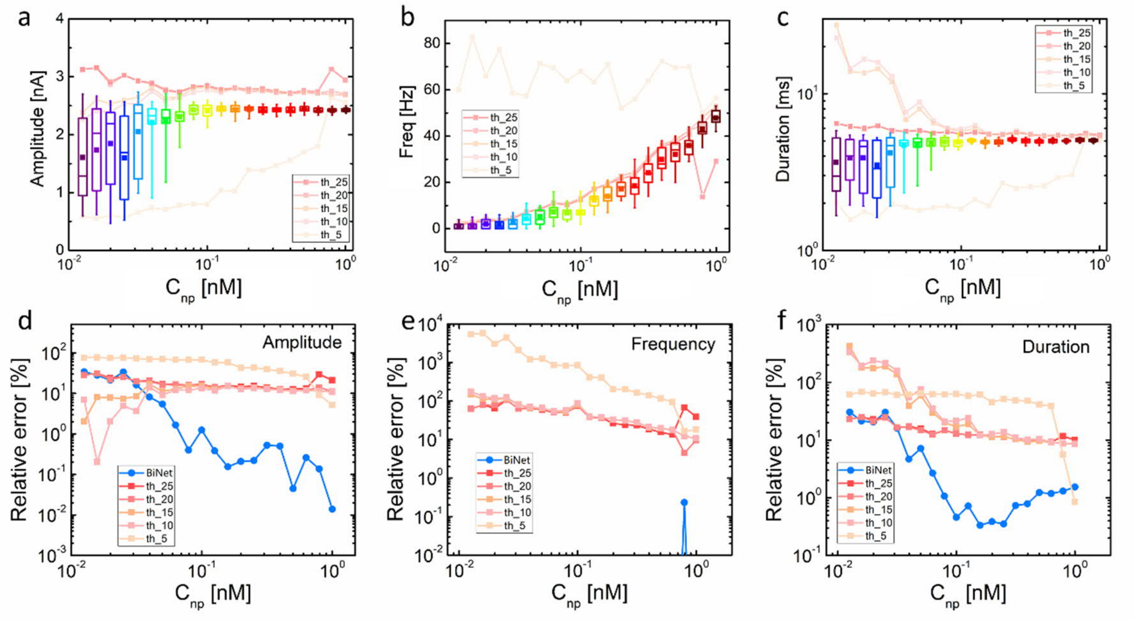

Figure S19. Comparison of the results from our neural network and those from the traditional algorithm on the dataset of varying C_{np}. (a-c) The box chart of the spike amplitude, frequency, and duration for translocating nanospheres with different concentration, C_{np}, respectively, extracted by our neural network. The average value of the spike amplitude, frequency, and duration extracted by the traditional algorithm with different thresholds are showed by the dot lines in corresponding figures for comparison. The relative error of the spike amplitude, frequency, and duration from our neural network and the traditional algorithm are compared in (c-f) respectively.

Artificially generated dataset, varying duration

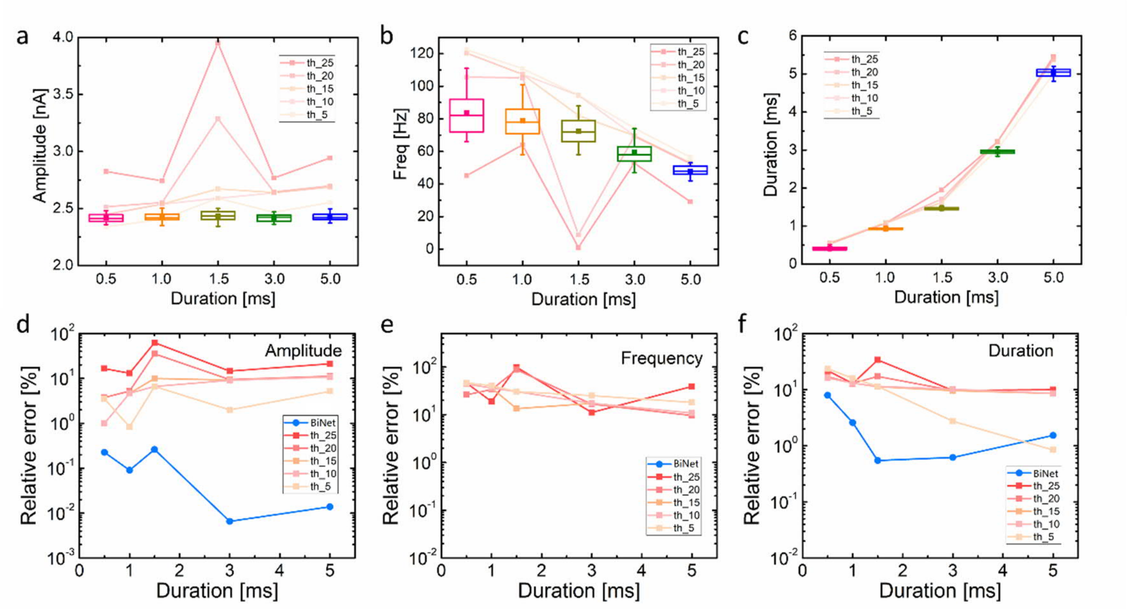

Figure S20. Comparison of the results from our neural network and those from the traditional algorithm on the dataset of varying duration. (a-c) The box chart of the spike amplitude,

frequency, and duration for translocating nanospheres with different duration, respectively, extracted by our neural network. The average value of the spike amplitude, frequency, and duration extracted by the traditional algorithm with different thresholds are showed by the dot lines in corresponding figures for comparison. The relative error of the spike amplitude, frequency, and duration from our neural network and the traditional algorithm are compared in (c-f) respectively.

Note 7. Translocation features of λ-DNA and *streptavidin* extracted by the B-Net

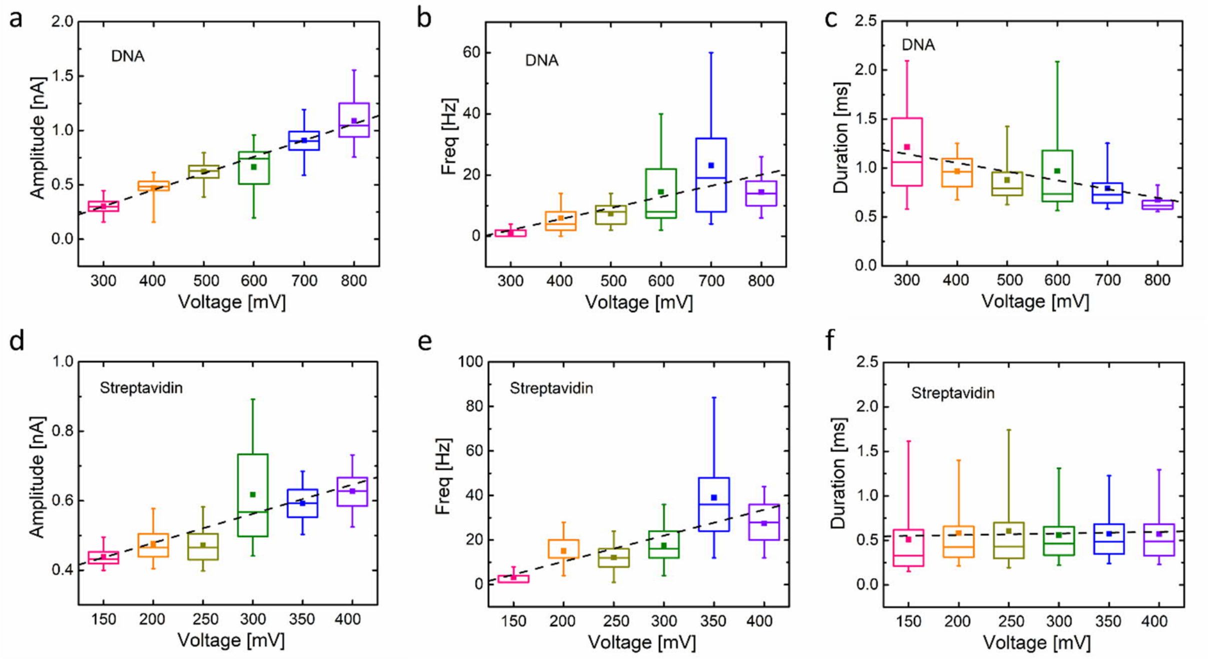

Figure S21. Signal processing of experimental data involving DNA and protein. Variation of spike amplitude, frequency and duration of λ-DNA (a-c) and *streptavidin* (d-f) translocation with bias voltage. All data are plotted in linear scale and linear regression is applied to each group of data, shown as the black dotted lines.

Note 8. Translocation frequency of *streptavidin*

According to Ref. 45, if the translocation kinetics is controlled by diffusion (*i.e.*, the translocation process is fast compared to the capture process), the theoretical translocation frequency of proteins should be

$$f = 2\pi c D r_p$$

where, c is the protein concentration [molecules/m^3], D the protein diffusivity in electrolyte [m^2/s], and r_p the radius of the nanopore [m]. In order to estimate the diffusivity of *streptavidin*, the following formula is adopted:[46]

$$D = \frac{6.85\times10^{-15} T}{\eta\sqrt{M^{1/3}R}}$$

where, T is temperature (300 [K]), η the viscosity of water (1×10^{-3} [Pa·s]), M the molecular weight of *streptavidin* (52.8×10^3 [Da]), and R the radius of *streptavidin* (3×10^{-9} [m]). Thus, the estimated diffusivity of *streptavidin* is 7.6×10^{-11} m^2/s, which is comparable to the reported diffusivity of other proteins (*cf.* Ref. 46 and SI of Ref. 45). Therefore, the expected translocation frequency is 193 Hz, with the concentration of 84 nM and nanopore radius of 8 nm. Referring to 193 Hz, the observation ratio at each bias voltage is calculated for the B-Net extracted results, as shown in Figure S22a. The theoretical translocation frequency is also marked in Figure S22b for better visualization.

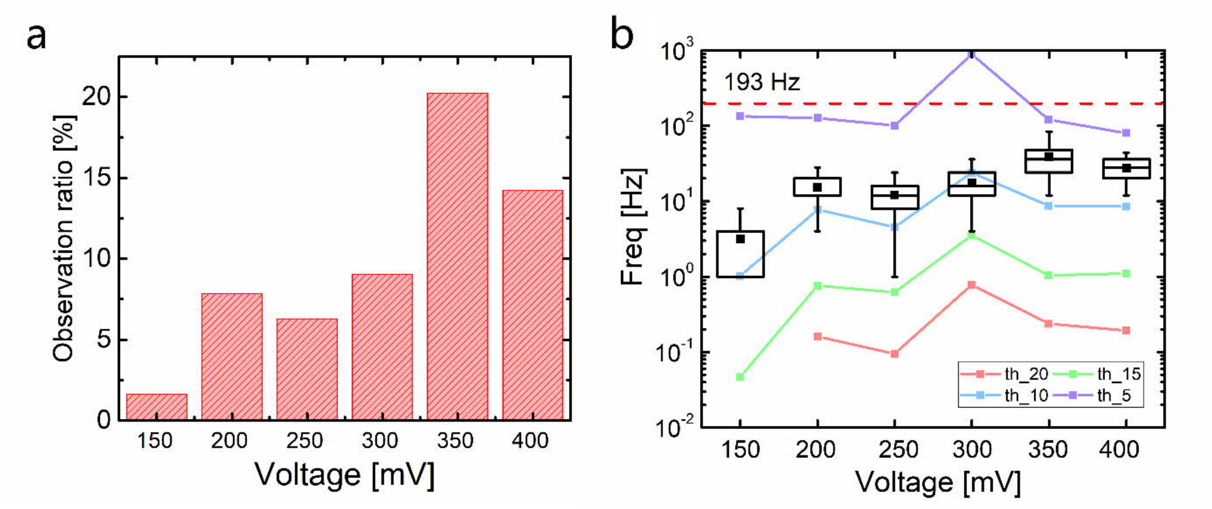

Figure S22. (a) Observation ratio of the *streptavidin* translocation events by the B-Net referring to the theoretical value. (b) Translocation frequency extracted by the B-Net (box chart) and traditional algorithm (dot lines). The theoretical frequency is marked by the horizontal red dash line.

The obtained observation ratio is reasonable compared to the results of translocation of other proteins (*cf.* Figure 2 of Ref. 45). It indicates that a certain amount of fast translocation events cannot be picked up by the electrical readout circuits, because of the limited bandwidth. However, on the other hand, we should also keep in mind that this model gives a simplified estimation holding the assumption that the protein translocation is only driven by the concentration gradient with a constant radius of the capture hemisphere (the physical radius of

nanopore). In reality, the capture sphere should increase with bias voltage.[36] In addition, the electroosmotic flow, *i.e.*, the convective transport, may also affect.

References

(1) Pedone, D.; Firnkes, M.; and Rant, U. Data Analysis of Translocation Events in Nanopore Experiments. *Anal. Chem.* **2009**, 81, 9689–9694.

(2) Raillon ,C.; Granjon, P.; Graf, M.; Steinbock , L.; Radenovic, A.; Fast and Automatic Processing of Multi-Level Events in Nanopore Translocation Experiments. *Nanoscale* **2012**, 4, 4916–4924.

(3) O'Donnell, C. R., Wiberg, Do. M.; Dunbar, W. B.; A Kalman Filter for Estimating Nanopore Channel Conductance in Voltage-Varying Experiments. *The 51st IEEE Conference on Decision and Control (CDC)*, IEEE **2012**, 2304–2309.

(4) Shekar, S.; Chien, C.-C.; Hartel, A.; Ong, P.; Clarke, O. B.; Marks, A.; Drndic, M.; Shepard, K. L. Wavelet Denoising of High-Bandwidth Nanopore and Ion-Channel Signals. *Nano lett.* **2019**, 19, 1090–1097.

(5) Zeng, S.; Wen, C.; Solomon, P.; Zhang, S.-L.; Zhang, Z. Rectification of Protein Translocation in Truncated Pyramidal Nanopores. *Nat. Nanotechnol*. **2019**, 14, 1056–1062.

(6) Balijepalli, A.; Ettedgui, J.; Cornio, A. T.; Robertson, J. W.F.; Cheung, K. P.; Kasianowicz, J. J.; Vaz, C. Quantifying Short-Lived Events in Multistate Ionic Current Measurements. *ACS Nano* **2014**, 8, 1547–1553.

(7) Gu, Z.; Ying, Y.-L.; Cao, C.; He, Pi.; Long, Y.-T. Accurate Data Process for Nanopore Analysis. *Anal. Chem.* **2015**, 87, 907–913.

(8) Im, J.; Sen, S.; Lindsay, S.; Zhang, P. Recognition Tunneling of Canonical and Modified RNA Nucleotides for Their Identification with the Aid of Machine Learning. *ACS Nano* **2018**, 12, 7067–7075.

(9) Larkin, J.; Henley, R. Y.; Muthukumar, M.; Rosenstein, J. K.; Wanunu, M. High-Bandwidth Protein Analysis Using Solid-State Nanopores. *Biophys. J.* **2014**, 106, 696–704.

(10) Sha, J.; Si, W.; Xu, B.; Zhang, S.; Li, K.; Lin, K.; Shi, H.; Chen, Y. Identification of Spherical and Nonspherical Proteins by a Solid-State Nanopore. *Anal. Chem.* **2018**, 90, 13826–13831.

(11) Tsutsui, M.; Yokota, K.; Arima, A.; He, Y.; Kawai, T. Solid-State Nanopore Time-of-Flight Mass Spectrometer. *ACS Sens*. **2019**, 4, 2974–2979.

(12) Houghtaling, J.; List, J.; Mayer, M. Nanopore-Based, Rapid Characterization of Individual Amyloid Particles in Solution: Concepts, Challenges, and Prospects. *Small* **2018**, 14, 1802412.

(13) Lan, W.-J.; Holden, D. A.; Zhang, B.; White, H. S. Nanoparticle Transport in Conical-Shaped Nanopores. *Anal. Chem*. **2011**, 83, 3840–3847.

(14) Houghtaling, J.; Ying, C.; Eggenberger, O. M.; Fennouri, A.; Nandivada, S.; Acharjee, M.; Li, J.; Hall, A. R.; Mayer, M. Estimation of Shape, Volume, and Dipole Moment of Individual Proteins Freely Transiting a Synthetic Nanopore. *ACS Nano* **2019**, 13, 5231–5242.

(15) Yusko, E. C.; Bruhn, B. R, Eggenberger, O. M.; Houghtaling, J.; Rollings, R. C. Walsh, N. C.; Nandivada, S.; Pindrus, M.; Hall, A. R.; Sept, D. D., Li, J.; Kalonia, D. S.; Mayer M. Real-Time Shape Approximation and Fingerprinting of Single Proteins Using a Nanopore. *Nat. Nanotechnol.* **2017**, 12, 360-367.

(16) Im, J.; Lindsay, S. L.; Wang, X.; Zhang, P. Single Molecule Identification and Quantification of Glycosaminoglycans Using Solid-State Nanopores. *ACS Nano* **2019**, 13, 6308–6318.

(17) Misiunas, K.; Ermann, N.; Keyser, U. F. QuipuNet: Convolutional Neural Network for Single-Molecule Nanopore Sensing. *Nano Lett.* **2018**, 18, 4040-4045.

(18) Wei, Z.-X.; Ying, Y.-L.; Li, M.-Y.; Yang, J.; Zhou, J.-L.; Wang, H.-F.; Yan, B.-Y.; Long, Y.-T. Learning Shapelets for Improving Single-Molecule Nanopore Sensing. *Anal. Chem.* **2019**, 91, 10033–10039.

(19) Arima, A.; Tsutsui, M.; Yoshida, T.; Tatematsu, K.; Yamazaki, T.; Yokota, K.; Kuroda, S.; Washio, T.; Baba, Y.; Kawai, T. Digital Pathology Platform for Respiratory Tract Infection Diagnosis *via* Multiplex Single-Particle Detections. *ACS Sens.* **2020**, 5, 3398–3403.

(20) Arima, A.; Tsutsui, M.; Washio, T.; Baba, Y.; Kawai, T. Solid-State Nanopore Platform Integrated with Machine Learning for Digital Diagnosis of Virus Infection. *Anal. Chem.* **2020**, 93, 215–227.

(21) Bengio, Y.; Courville, A.; Vincent, P. Representation Learning: A Review and New Perspectives. *IEEE Trans. Pattern Anal. Mach. Intell.*, **2013**, 35, 1798–1828.

(22) LeCun, Y.; Bengio, Y.; Hinton, G. Deep Learning. *Nature* **2015**, 521, 436–444.

(23) Schmidhuber, J. Deep Learning in Neural Networks: An Overview. *Neural Networks* **2015**, 61, 85–117.

(24) He, K.; Zhang, X.; Ren, S.; Sun, J. Deep Residual Learning for Image Recognition. **2015**, 1512.03385. *arXiv*. https://arxiv.org/abs/1512.03385 (accessed Feb. 1st, 2021).

(25) He, K.; Zhang, X.; Ren, S.; Sun, J. Deep Residual Learning for Image Recognition. *IEEE Conference on Computer Vision and Pattern Recognition (CVPR)*, IEEE **2016**, 770–778.

(26) Wen, C.; Zhang, Z.; Zhang, S.-L. Physical Model for Rapid and Accurate Determination of Nanopore Size *via* Conductance Measurement. *ACS Sens.* **2017**, 2, 1523-1530.

(27) Anderson, B. N.; Muthukumar, M.; Meller, A. *p*H Tuning of DNA Translocation Time through Organically Functionalized Nanopores. *ACS Nano* **2013**, 7, 1408–1414.

(28) Kox, R.; Deheryan, S.; Chen, C.; Arjmandi, N.; Lagae, L.; Borghs, G. Local Solid-State Modification of Nanopore Surface Charges. *Nanotechnology* **2010**, 21, 335703.

(29) Luan, B.; Stolovitzky, G. An Electro-Hydrodynamics-Based Model for the Ionic Conductivity of Solid-State Nanopores during DNA Translocation. *Nanotechnology* **2013**, 24, 195702.

(30) Adamson, A. Chapter 13. Electrochemical Cells. In *A Textbook of Physical Chemistry*, page 506. Academic Press, New York, 1ed, **1973**.

(31) Wen, C.; Zeng, S.; Zhang, Z.; Hjort, K.; Scheicher, R.; Zhang, S.-L. On Nanopore DNA Sequencing by Signal and Noise Analysis of Ionic Current. *Nanotechnology* **2016**, 27, 215502.

(32) Wen, C.; Zeng, S.; Zhang, Z.; Zhang, S.-L. Group Behavior of Nanoparticles Translocating Multiple Nanopores. *Anal. Chem.* **2018**, 90, 13483-13490.

(33) Wen, C.; Zeng, S.; Arstila, K.; Sajavaara, T.; Zhu, Y.; Zhang, Z.; Zhang, S.-L. Generalized Noise Study of Solid-State Nanopores at Low Frequencies. *ACS Sens.* **2017**, 2, 300-307.

(34) Smeets, R. M. M.; Dekker, N. H.; Dekker, C. Low-Frequency Noise in Solid-State Nanopores. *Nanotechnology* **2009**, 20, 095501.

(35) Smeets, R. M.; Keyser, U. F.; Dekker, N. H.; Dekker, C. Noise in Solid-State Nanopores. *Proc. Natl. Acad. Sci. U.S.A.* **2008**, 105, 417-421.

(36) Wen, C.; Zhang, S.-L. Fundamentals and Potentials of Solid-State Nanopores: A Review. *J. Phys. D: Appl. Phys.* **2020**, 54, 023001.

(37) Liu, B.; Liu, J. Overview of Image Denoising Based on Deep Learning. *J. Phys. Conf. Ser.* **2019**, 1176, 022010.

(38) Tian, C.; Fei, L.; Zheng, W.; Xu, Y.; Zuo, W.; Lin, C.-W. Deep Learning on Image Denoising: An Overview. **2020**, 1912.13171. *arXiv*. https://arxiv.org/abs/1912.13171 (accessed Feb. 1st, 2021).

(39) Ivanov, T.; Kumar, A.; Sharoukhov, D.; Ortega, F.; Putman, M. DeepDenoise: A Deep Learning Model for Noise Reduction in Low SNR Imaging Conditions. *Proceedings of SPIE*. **2020**, 11511, 20–28.

(40) Jin, L.; Zhang, W.; Ma, G.; Song, E. Learning Deep CNNs for Impulse Noise Removal in Images. *J. Vis. Commun. Image Represent.* **2019**, 62, 193–205.

(41) Serizel, R. A Brief Introduction to Multichannel Noise Reduction with Deep Neural Networks. *SpiN 2020-12th Speech in Noise Workshop*, France, SpiN **2020**. hal-02506387.

(42) Kumar A.; Florencio, D. Speech Enhancement in Multiple-Noise Conditions Using Deep Neural Networks. 2016, 1605.02427. *arXiv*. https://arxiv.org/abs/1605.02427 (accessed Feb. 1st, 2021).

(43) Lai, Y.-H.; Tsao, Y.; Lu, X.; Chen, F.; Su, Y.-T.; Chen, K.-C.; Chen, Y.-H.; Chen, L.-C.; Li, L. P.-H.; Lee, C.-H. Deep Learning-Based Noise Reduction Approach to Improve Speech Intelligibility for Cochlear Implant Recipients. *Ear Hear.* **2018**, 39, 795–809.

(44) Yu, S.; Tomasi, C. Identity Connections in Residual Nets Improve Noise Stability. **2019**, 1905.10944. *arXiv*. https://arxiv.org/abs/1905.10944 (accessed Feb. 1st, 2021).

(45) Plesa, C.; Kowalczyk, S. W.; Zinsmeester, R.; Grosberg, A. Y.; Rabin, Y.; Dekker, C. Fast Translocation of Proteins through Solid State Nanopores. *Nano Lett.* **2013**, 13, 658-663.

(46) He, L.; Niemeyer, B. A Novel Correlation for Protein Diffusion Coefficients Based on Molecular Weight and Radius of Gyration. *Biotechnol. Prog.* **2003**, 19, 544–548.
